# Using pseudotime derivative on single-cell RNA sequencing data to identify genes undergoing cell cycle regulation

**DOI:** 10.1093/bioadv/vbaf123

**Published:** 2025-05-29

**Authors:** Yohan Lefol, Geir Amund Svan Hasle, Siv Anita Hegre, Helle Samdal, Pål Sætrom

**Affiliations:** Department of Clinical and Molecular Medicine, NTNU—Norwegian University of Science and Technology, Trondheim, NO-7491, Norway; Génie Physiologique, Biotechnologique et Informatique, Université de Poitiers, 8600, Poitier, France; Department of Microbiology, Institute of Clinical Medicine, University of Oslo, Oslo, NO-0373, Norway; CRESCO, Centre for Embryology and Healthy Development, University of Oslo, Oslo, NO-0373, Norway; Department of Clinical and Molecular Medicine, NTNU—Norwegian University of Science and Technology, Trondheim, NO-7491, Norway; St. Olavs Hospital HF, Sentral Stab, Trondheim, NO-7006, Norway; Department of Clinical and Molecular Medicine, NTNU—Norwegian University of Science and Technology, Trondheim, NO-7491, Norway; Department of Clinical and Molecular Medicine, NTNU—Norwegian University of Science and Technology, Trondheim, NO-7491, Norway; Department of Clinical and Molecular Medicine, NTNU—Norwegian University of Science and Technology, Trondheim, NO-7491, Norway; St. Olavs Hospital HF, Sentral Stab, Trondheim, NO-7006, Norway; Department of Computer Science, NTNU—Norwegian University of Science and Technology, Trondheim, NO-7491, Norway; K.G. Jebsen Center for Genetic Epidemiology, NTNU—Norwegian University of Science and Technology, Trondheim, NO-7491, Norway

## Abstract

**Motivation:**

The cell cycle is a critical part of cellular life, one that has long been studied, both directly, and through its regulatory components. Commonly, cell cycle synchronization or selection experiments are performed in order to study the cell cycle, thus chemically modifying the cells, or selecting them for specific phases. We seek to develop a means to study the cell cycle through the use of single cell RNA sequencing, effectively circumventing the need for such experiments.

**Results:**

We utilize a well-established pseudotime method, along with the predicted and real expression of genes to calculate the velocity of individual genes. We then utilize statistics and expected biological behaviour to identify genes with significant shifts in velocity within the pseudotime. Additionally, we show the ability to observe gene regulatory behaviour such as mRNA splicing and degradation rates. As many cell line based research utilize multiple replicates we implement a merger method for technical replicates to adjust for technical variations, creating a more robust analysis. In summary, our study develops a robust approach to map the velocities of individual, biologically, and statistically significant genes throughout the cell cycle’s phases within a cell line experiment.

**Availability and implementation:**

Data and code are available at: https://github.com/Ylefol/CC_vel.

## 1 Introduction

The cell cycle is a critical component for most living organisms. In order to study the cell cycle, cell synchronization is commonly utilized (Futcher 1999). Cell synchronization often involves the use of a variety of chemicals to synchronize all the cells of a sample to a specific phase of the cell cycle. A commonly cited disadvantage of this approach is that the environment must first be modified in order to synchronize the cells, which in turn may affect the results of downstream analyses (Cooper 2019). There are methods to study the cell cycle without the utilization of cell synchronization, but these typically involve the isolation of cells at specific points of the cell cycle. Such methods include fluorescence activated cell sorting, size separation, or collecting recently divided cells. Whereas these methods do not rely on chemical perturbations of the cells, the selection methods can potentially affect gene expression and cellular state through mechanical stress (Box *et al.* 2020, Ryan *et al.* 2021, Wagh *et al.* 2021). Finally, both synchronization and selection methods can provide data at specific states of the cell cycle; however, these methods cannot easily be used to study cells through the continuous stages of the cell cycle.

One solution to the study of an entire population of cells within a sample is the use of single cell RNA sequencing (scRNA-seq). Early scRNA-seq studies have found that cells undergoing cell division can form sub-clusters within their respective cell types (Scialdone *et al.* 2015, Chen and Zhou 2017), thus showing that scRNA-seq has the ability to identify subsets of dividing cells based on gene expression. Furthermore, Scialdone et al. showed that PCA-based classifications gave the most accurate phase assignment results, which was also confirmed by Kowalczyk *et al.* (2015) and Tirosh *et al.* (2016). This has been further confirmed by experiments utilizing fluorescent ubiquitination cell cycle indicators (FUCCI) to validated cell cycle phase assignment (Hsiao *et al.* 2020). DeepCycle is a machine learning based tool created to perform the same task; establishing a cell cycle pseudotime and subsequently assign cell cycle phases to individual cells (Riba *et al.* 2022). To then study the dynamics of the cell cycle, including ordering cells based on their cell cycle progression, the concept of RNA velocity may be used.

RNA velocity is a term coined to describe changes in a cell’s gene expression that can predict the cell’s future state (La Manno *et al.* 2018). The basis of RNA velocity comes from the calculation of the relative abundance of precursor messenger RNA (pre-mRNA) as well as mature mRNA. Currently, there are two main methods that calculate RNA velocity from scRNA-seq data: Velocyto and scVelo. Both are based on the same framework of differential equations describing the relationship between pre-mRNA and mRNA levels. The primary difference between these two methods is their assumptions pertaining to transcriptional induction and repression. Velocyto’s steady state model assumes that all genes share a common splicing rate and that the complete dynamics of gene expression are captured by the sequencing data (La Manno *et al.* 2018). scVelo’s dynamic model assumes that these events are dynamic (Bergen *et al.* 2020). Both methods begin by calculating the velocity of individual genes, which is then utilized to extrapolate a cell’s fate, including creating a pseudotime ordering of the cells.

Whereas Velocyto and scVelo can provide data-driven characteristics of cellular dynamics without any prior information, the known biology of the cell cycle, such as the order of cell cycle phases, should be taken into account when studying cell cycle dynamics. We therefore propose an approach adapted from the method presented by Scialdone et al.; Each cell is given a cell cycle score based on their expression of 172 validated cell cycle genes (Whitfield *et al.* 2002, Kowalczyk *et al.* 2015, Tirosh *et al.* 2016), from these scores angle boundaries for each cell cycle phase can be drawn within a PCA, cells can then be re-assigned to a specific cell cycle phase based on their location in the PCA rather than their cell cycle score. By having clear phase transition points we are able to establish a pseudotime with a clearly defined ‘start’, in this case, the S/G2M transition point. Following the establishment of the pseudotime and cell cycle phase boundaries, we compute RNA velocity of individual genes, identify genes with significant expression changes through the cell cycle, and characterize genes based on the pseudotime delays between their pre-mRNA and mRNA expression profiles. In this aspect, we utilize both statistics and expected biological behaviour of pre-mRNA levels before or at the same time as mRNA levels to identify genes with cell cycle dependant behaviour. We demonstrate our method on scRNA-seq data from unsynchronized human keratinocytes and publicly available data from Jurkat and 293T cells and show highly significant overlaps between significant cell cycle genes in the three cell lines. Furthermore, these genes are highly enriched for known cell cycle functions and for genes with increased expression in solid tumours from the cancer genome atlas (TCGA) study. Our proposed method is available as a snakemake pipeline with an associated tutorial at https://github.com/Ylefol/CC_vel.

## 2 Materials and methods

Detailed materials and methods can be found in File 1, available as supplementary data at *Bioinformatics Advances* online.

## 3 Results and discussion

**Figure 1.**
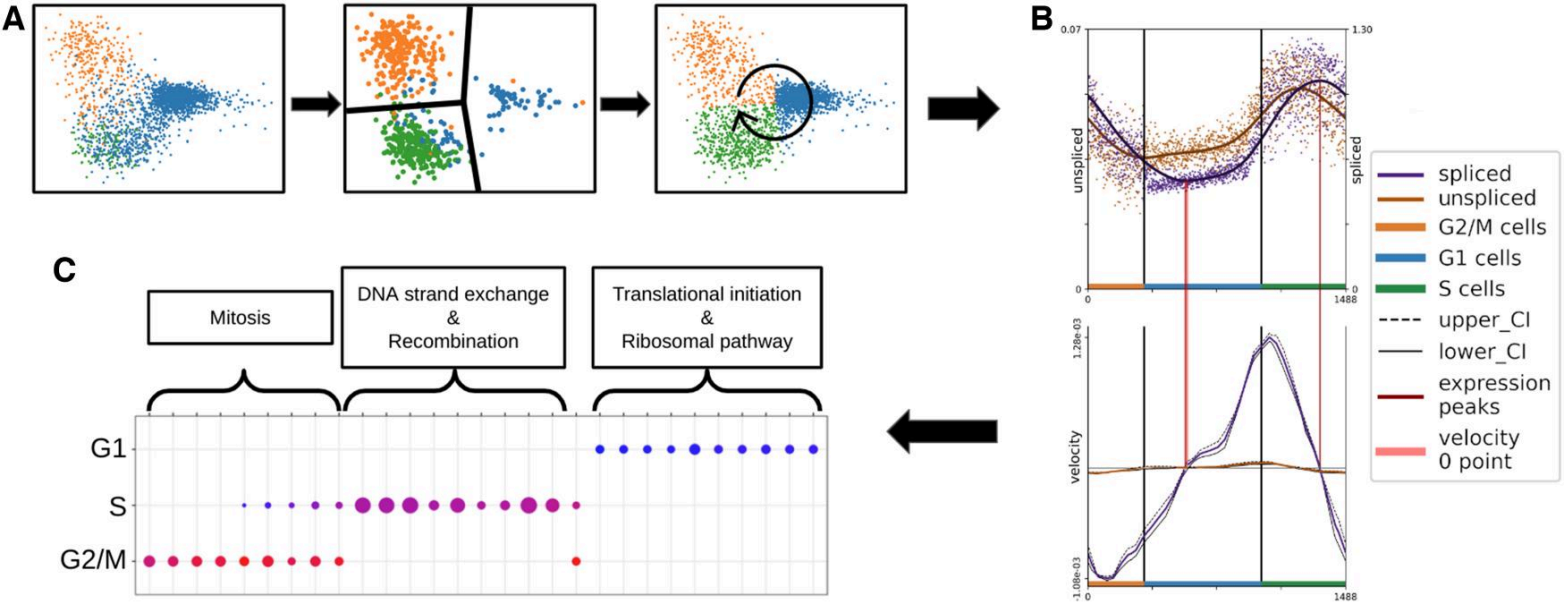
A conceptual figure illustrating the overall workflow contained within this manuscript. (A) Depicts a series of three PCA plots showing the initial cell cycle phase assignment of individual cells, to the filtering process and establishment of cell cycle boundaries, ending with the reassignment of cell cycle phases based on the calculated boundaries. The last plot also illustrates the established cell cycle pseudotime beginning at the S-G2/M boundary due to its consistency across cell lines. Blue dots represent G1 cells, green dots are S cells and orange represents G2/M cells. (B) Illustrates the calculation of an individual genes velocity based on its spliced and unspliced expression data. The above plot shows a genes spliced expression and unspliced expression where points are cells and lines are means, while the bottom plot shows the velocities and confidence intervals (CI) calculated from the expression. Genes which have a positive and negative velocity are submitted to a *t*-test to determine their significance. (C) Shows the over-representation results when associating significant genes to cell cycle phases based on peak spliced expression. The dotplot shows strong cell cycle phase related pathways for both S and G2/M while showing weak house-keeping pathways for G1. This shows that genes with significant shifts in velocity are mostly related to cell cycle pathways.

In scRNA-seq data, genes with known cell cycle-dependent expression can be used to cluster cells into groups representing distinct phases of the cell cycle (Scialdone *et al.* 2015, Chen and Zhou 2017). This clustering can be readily visualized by plotting cells as a function of the first and second principal components (PC1 and PC2) in a principal component analysis (PCA) of these genes (Fig. 1A). For periodic phase-shifted data, PC1 and PC2 represent a sine, cosine decomposition of the data; the angle in the corresponding plane can then be used to order the data based on their phase.

Here, we present a method that uses the above-mentioned properties to analyse the dynamics of gene expression in scRNA-seq data of asynchronously dividing cells. Specifically, we (i) use the PCA phase angle to establish a cell cycle pseudotime ordering of the cells, (ii) use this pseudotime ordering to compute pre-mRNA and mRNA gene expression profiles and velocities, (iii) identify genes with significant expression changes, and (iv) evaluate their biological functions (Fig. 1). Our method is implemented as a snakemake pipeline consisting of quality control, establishing cellular pseudotime, computing gene velocity, combining technical replicates, identifying genes with significant non-zero velocity during the cell cycle, computing pseudotime delays between pre-mRNA and mRNA profiles, calculating a cell cycle variance threshold, and evaluating functional enrichment of significant genes. The following sections describe these steps and show the results of using our method on scRNA-seq data from three distinct cell lines: data from human keratinocytes (HaCaT) generated for this study, and publicly available data from Jurkat and 293T cells. We also include results of a HeLa dataset as further validation.

### 3.1 Quality control

The HaCaT cell line is genomically stable and has high differentiating and proliferating capacity *in vitro* (Wilson 2014); we have previously used this cell line to study cell cycle-dependent gene expression by cell synchronization (Pena-Diaz *et al.* 2013, Hegre *et al.* 2021). Therefore, to test our proposed method for cell cycle analyses of scRNA-seq data, we generated scRNA-seq data from asynchronously proliferating HaCaT cells, mixed with mouse embryonic fibroblasts at a 9:1 ratio for experimental quality control. Following sequencing and data quality control, our dataset consisted of two technical replicates with 2399 and 2085 cells and 16 181 and 16 002 expressed genes, respectively (Table 1, available as supplementary data at *Bioinformatics Advances* online).

### 3.2 Establishing a pseudotime order

**Figure 2.**
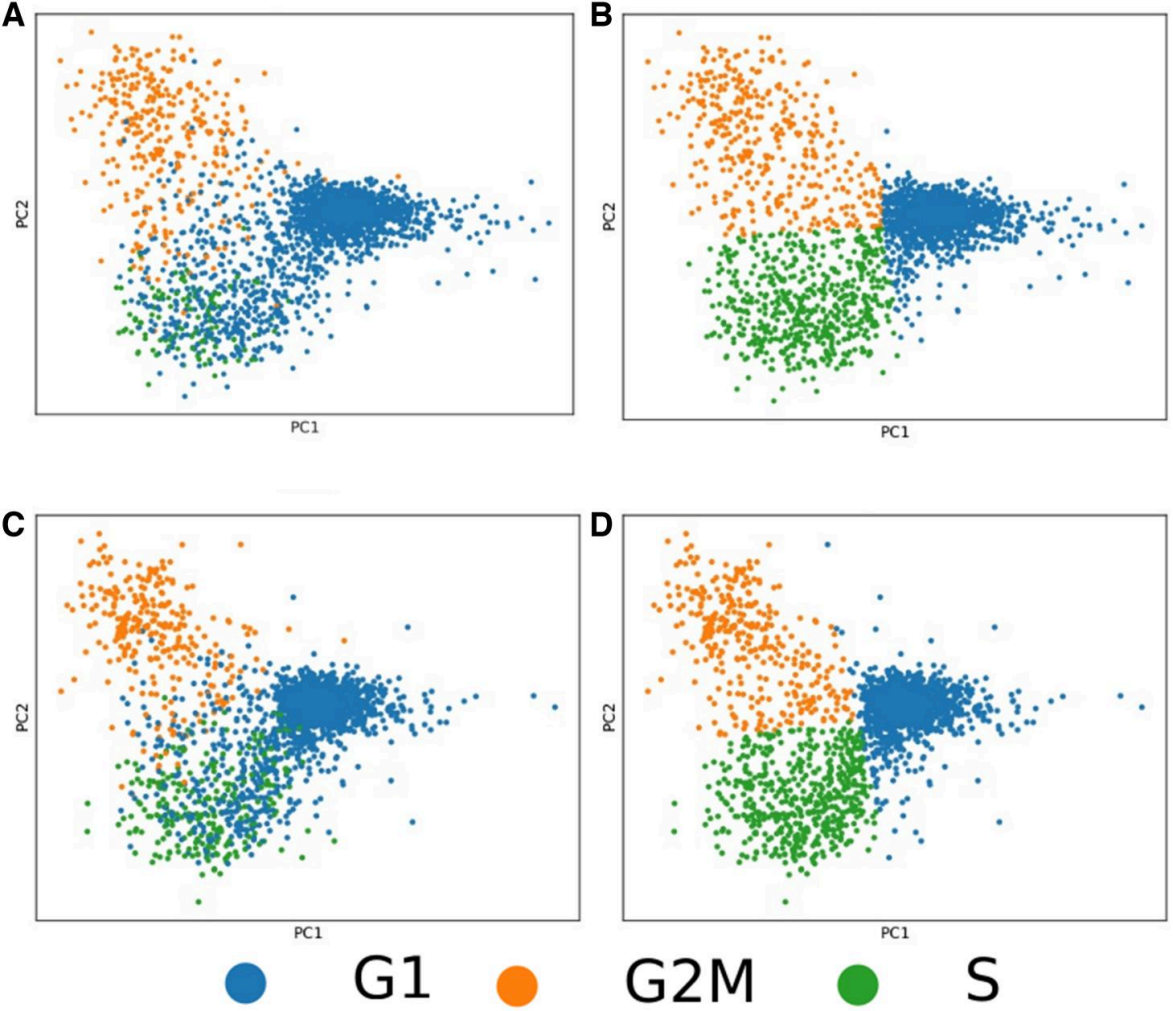
PCA plots represent the process of phase reassignment for both replicates. n_counts (counts per cell before normalization) was regressed out for both replicates before the reassignment (A, C). Following the identification of phase boundaries, cells are reassigned to a cell cycle phase (B, D). Each point represents a cell.

After selecting genes with known cell cycle-dependent expression (Whitfield *et al.* 2002, Kowalczyk *et al.* 2015), 172 genes remained in each replicate. PCA plots of the resulting data show cells clustering based on their individually assigned cell cycle phases in a pattern that was very similar between the two technical replicates (Fig. 2). Notably, the predicted cell cycle phases were less distinct for cells around the G1-S transition point compared to cells predicted to be in G2/M. As the number of known genes associated with the G1 phase is small compared with those associated with the S and G2/M phases [eight compared with 74 and 90 genes, respectively; Whitfield et al. (2002)], the G1 predictions can be more susceptible to outliers or missing values in the data. Although the number of G1 outliers is small, we performed a quick comparison between G1 cells within the S cluster with the cells in the G1 cluster, this is repeated with the G2M cluster. We isolated differentially expressed genes for the G1 cells in each respective cluster (File 2, available as supplementary data at *Bioinformatics Advances* online). We then inputted the two list to gprofiler (Raudvere *et al.* 2019). The genes for the G1 cells in the S cluster mapped to cell cycle related REAC terms, notably the terms ‘S phase’ and ‘G1/S transition’ (Table 2, available as supplementary data at *Bioinformatics Advances* online). The genes of the G1 cells in G2M primarily mapped to mitotic processes (Table 3, available as supplementary data at *Bioinformatics Advances* online). These results indicate that the infiltrating G1 associated cells are indeed where they belong, in the case of the S cluster these cells seem to partially mark the transition from G1 to S, though are primarily associated to S. Nonetheless we used the phase angle distributions of the top-scoring cells assigned to the S and G2/M phases to establish phase boundaries in the pseudotime ordering. Similarly, due to its more robust identification, we used the S-G2/M boundary as pseudotime 0.

Note that a general assumption made during this study is that the majority of the cells do not enter G_0_. This assumption is backed by the PCA results (Fig. 2), where we do not distinguish a group of cells beyond the three cell cycle phases searched for. G_0_ cells would be expected to be near the G1 cluster. An argument could be made that a small number of G1 associated cells located on the far right-hand side of the PCA could be a separate group, though there is no method to distinguish between G1 and G_0_ cells via gene expression alone. Nonetheless, this small group remains inconsequential based on the total number of cells found within the dataset.

### 3.3 Gene expression along a cell cycle pseudotime

**Figure 3.**
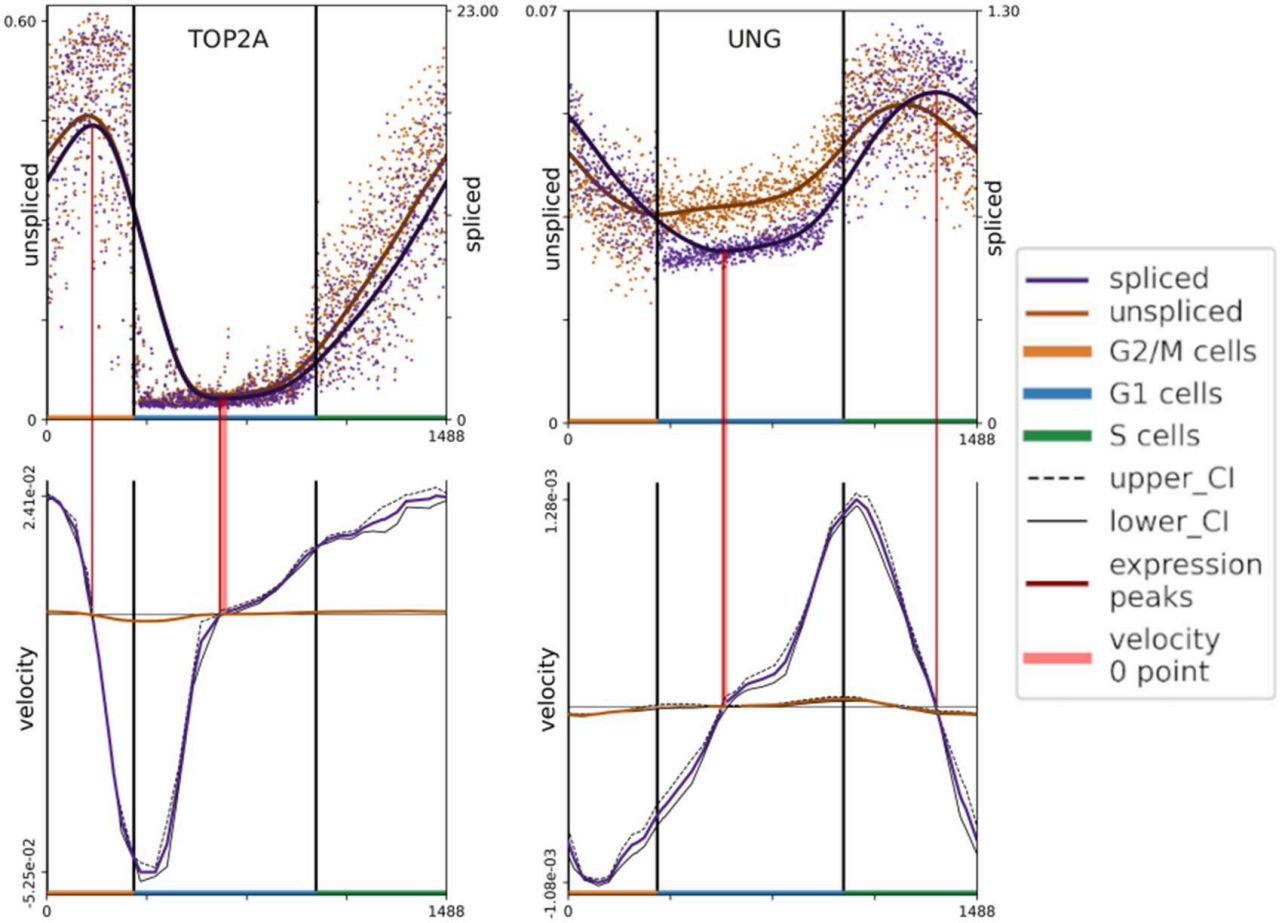
Expression (top) and velocity (bottom) of the *TOP2A* (left) and *UNG* (right) genes for the HaCaT cell line with merged replicates. The expression data are shown in the upper plot where individual dots show the expression of individual cells while the thicker lines show the smoothed expression data. The velocities are seen in the lower plots. Upper confidence intervals are shown as dashed lines while lower confidence intervals are solid lines. Red lines between the upper and lower plots show the location of peaks (upper and lower) in spliced expression. The red line shows that the expression peaks mark when velocity shifts from positive to negative or vice versa.

Based on the pseudotime ordering from the PCA analysis, we compute normalized pre-mRNA and mRNA expression profiles and velocities, illustrated in Fig. 3 for the gene uracil-DNA glycosylase (*UNG*) and DNA Topoisomerase II Alpha (*TOP2A*). The pre-mRNA velocity serves as a ‘prediction’ for the mRNA expression, where positive velocity represents an increase in active transcription while negative velocity represents a decrease in transcription.

The *UNG* gene plays an important role in DNA damage repair and is expected to be highly active in S phase (Slupphaug *et al.* 1991, Hagen *et al.* 2008, Tirosh *et al.* 2016). In our results, active *UNG* transcription (positive velocity) starts in mid-G1 phase, peaks in S phase, and ends (negative velocities) before the end of S phase (Fig. 3). These results are consistent with (Mjelle *et al.* 2015), which found that *UNG* peaks in late G1/early S while also declining within S phase in data from synchronized cells. *TOP2A* plays a wide variety of roles, one particular role seems to be the adjustment of DNA topology during the mitotic stage of the cell cycle, where the absence of TOP2A results in an early exit of Mitosis (Nielsen *et al.* 2020). Along with this, TOP2A, along with TOP2B, is known to play an important role in the proliferation of a variety of cancers (Uusküla-Reimand and Wilson 2022). Put together, this implicates TOP2A as a gene which is highly active in G2M, this is correctly represented by our results (Fig. 3).

### 3.4 Comparison with DeepCycle

We compare our adapted pseudotime and phase assignment method with DeepCycle (Riba *et al.* 2022). DeepCycle identified all three phase transitions (M/G1, G1/S, S/G2) in HaCaT A (Fig. 1, available as supplementary data at *Bioinformatics Advances* online) but failed to detect the M/G1 transition in HaCaT B. DeepCycle determines M/G1 by identifying the first transcriptional phase bin where RNA counts (UMI) per cell drop, a method designed for cell populations rather than cell lines, which may explain its failure in HaCaT B. For HaCaT A, where the transition was detected, DeepCycle’s phase assignments differ markedly from ours (Figs 2 and 3, available as supplementary data at *Bioinformatics Advances* online). It assigns TOP2A’s peak to G1 instead of G2M (Nielsen *et al.* 2020, Uusküla-Reimand and Wilson 2022) and UNG’s peak correctly to late G1/early S but inaccurately shows its expression increasing in late S instead of G1 (Mjelle *et al.* 2015). These results suggest that while DeepCycle generates a pseudotime, it fails at accurate phase assignment and only works for one of our two HaCaT replicates. Thus, we do not use DeepCycle moving forward.

### 3.5 Merging technical replicates

**Figure 4.**
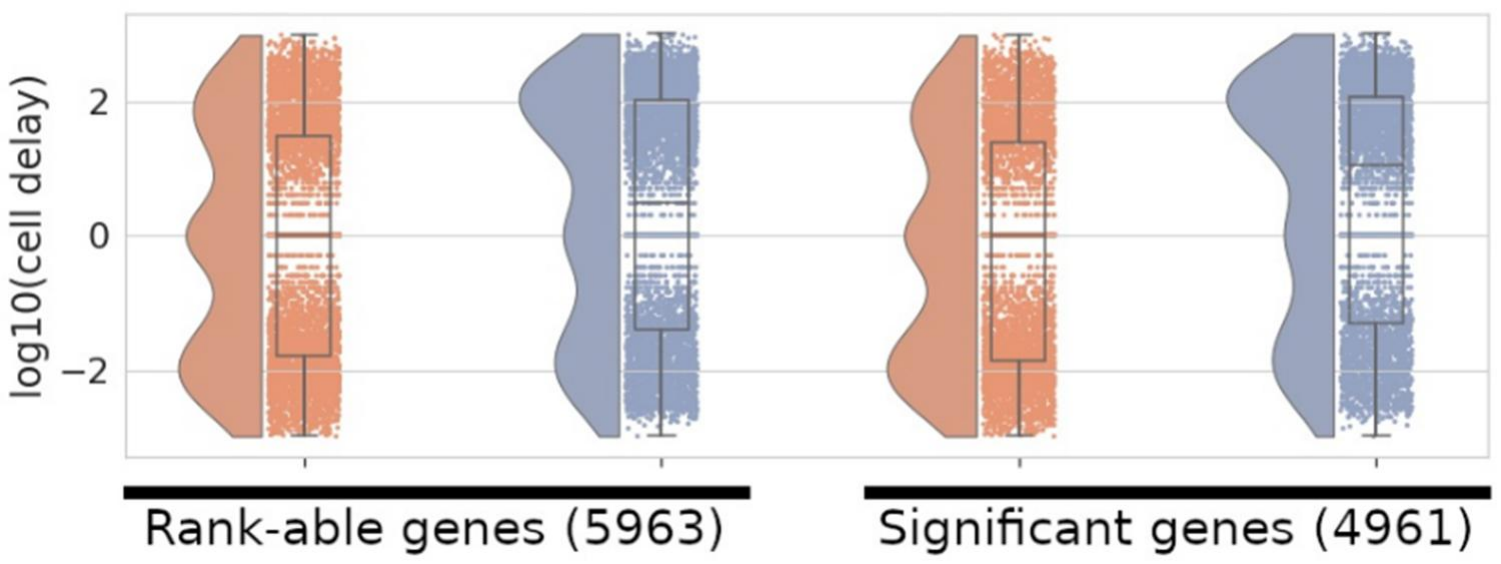
Raincloud plot showing two delay categories using two different sets of genes. Orange shows the ‘increase to 1’ and blue shows ‘decrease to 0’. Significant genes are genes with an adjusted *P* value (from a *t*-test) below 0.01. These plots show boxplots, violin plots, and jitter plot of the log10 transformed delay values for each category.

**Figure 5.**
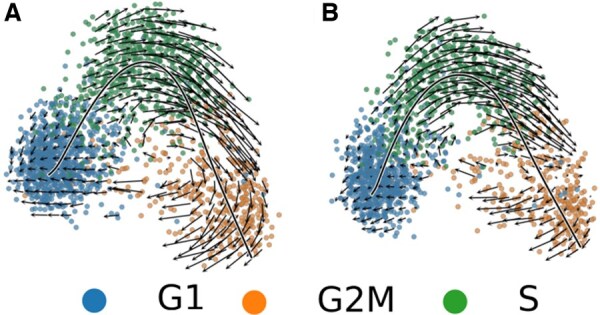
The velocity fields for both technical replicates, the field represents the different phases of the cell cycle and the expected differentiation path based on gene expression. The principal curve shows the overall directionality of the data points. Each point in the velocity field represents an individual cell.

When comparing the technical replicates in the HaCaT data, we noted some variation between spliced and unspliced velocities, as seen in the velocities for the *UNG* and *TOP2A* genes (Fig. 4, available as supplementary data at *Bioinformatics Advances* online). To accommodate our analyses to variation between replicates, we adapted the method in order to use replicates to determine which areas of a gene’s velocity is consistent between replicates thus increasing the robustness of the method. This is illustrated by 99% CI for each gene’s velocity curves (Fig. 5, available as supplementary data at *Bioinformatics Advances* online).

### 3.6 Determining gene significance and delay

Genes with cell cycle-dependent gene expression should have both increasing and decreasing expression throughout the cell cycle pseudotime. To identify genes of interest, we, therefore, start by filtering for genes which exhibit both positive and negative velocity, which in the HaCaT data, resulted in 5963 genes which we term ‘rank-able’. We then used a Student’s *t*-test on the gene’s peak mRNA velocity to identify significant genes, which, after correcting for multiple testing, identified 4961 significant genes (adjusted P<0.01).

To further characterize the expression dynamics of these genes, we measured the pseudotime delay for the rank-able and significant genes (Fig. 4). Delays are defined as the pseudotime difference between mRNA and pre-mRNA velocities, where a positive delay indicates that a change in pre-mRNA velocity occurred before the same change in mRNA velocity. Negative delay values indicate the opposite.

To be consistent with a model where changes in pre-mRNA levels are followed by changes in mRNA levels, gene-level delays should be zero or positive. This was the case for most of the rank-able and significant genes in our results when considering the delay between maximum mRNA and pre-mRNA expression [‘decrease to 0’ (blue) in Fig. 4], where both curves are likely to be most accurate. In contrast, the category illustrating the beginning of active transcription to peak velocity [increase to +1 (orange)] shows that most genes had negative delays between mature mRNA and pre-mRNA expression indicating that the prediction potential of pre-mRNA in regards to mRNA is stronger when expression heads towards, and reaches, its peak.

Based on the assumption that genes with cell-cycle-dependent expression should all have zero or positive delays, we sought to identify the genes that had significant negative delays, thereby violating the transcriptional model’s assumptions. To identify the genes with significant negative delays, we modelled the empirical delay distribution as a Gaussian mixture model to split the trimodal delay distribution into three normally distributed curves, we then determined the 95th percentile of the normal distribution with a mean value of, or closest to 0, and used that value as a negative delay threshold (Fig. 5, available as supplementary data at *Bioinformatics Advances* online).

Using this method we identified several significant negative delay genes, specifically 170 for HaCaT, 1000 for 293T, and 590 for Jurkat (File 1, available as supplementary data at *Bioinformatics Advances* online). In order to investigate if these genes were related to a biological condition that our pseudotime did not accommodate, we performed an over-representation analysis with each gene list (Tables 4–6, available as supplementary data at *Bioinformatics Advances* online). These analyses revealed no consistency between cell lines thus indicating that the likely cause of these significant negative delays is due to technical artefacts. We therefore validate the need to filter out these genes. Although we utilize these delays to filter out technical artefacts, we hypothesize that they may be used in a wider application. They could also provide insights into a gene’s post-transcriptional regulatory mechanisms via its rate of pre-mRNA splicing and mRNA degradation. Specifically, an increase in the rate of mRNA splicing would create an increased distance between peak pre-mRNA and peak mRNA expression, whereas an increase in the rate of mRNA degradation would decrease the same distance. Consequently, the genes with small delays in peak expression may have distinct post-transcriptional regulatory mechanisms from those with longer delays.

### 3.7 Comparing significant genes between cell lines

**Figure 6.**
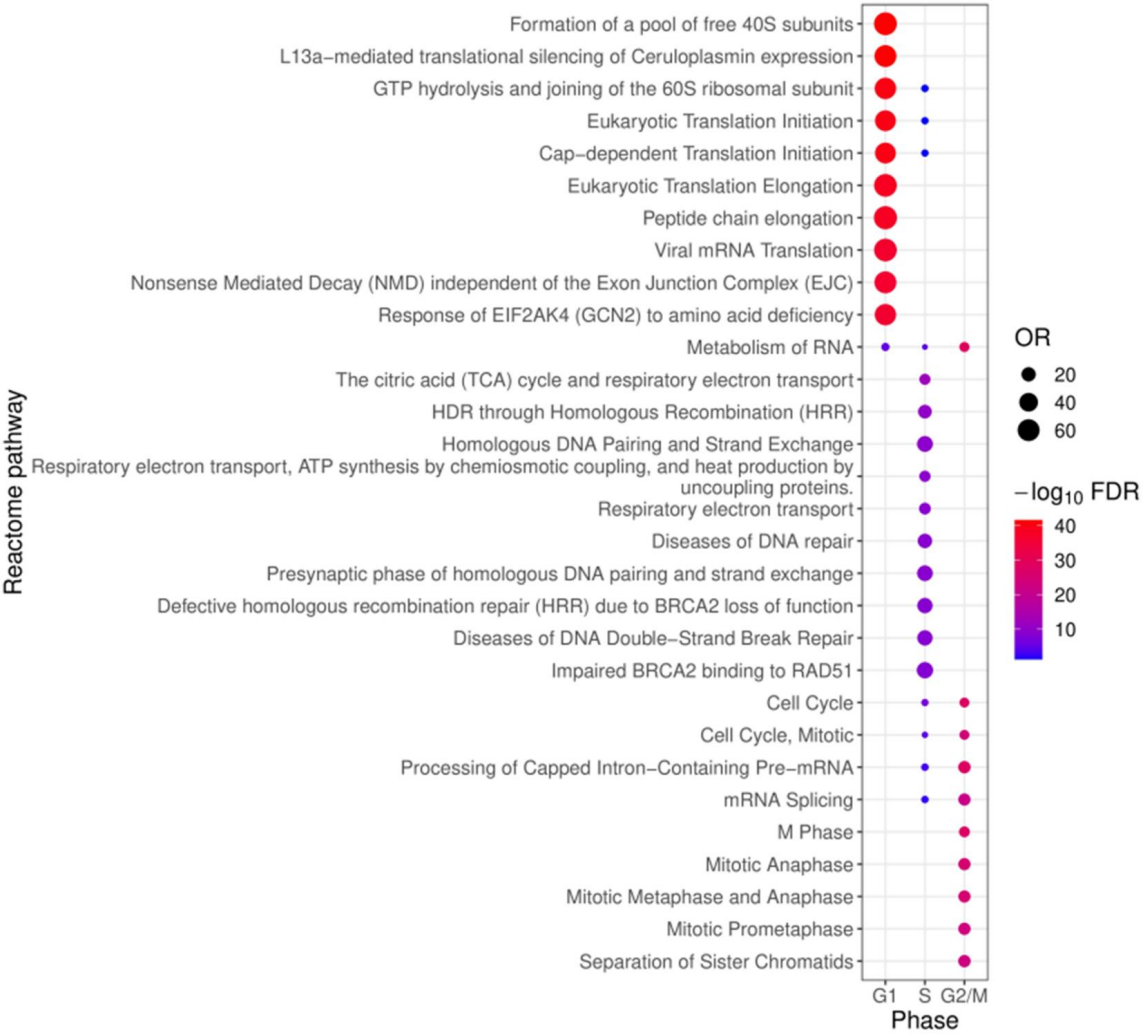
A dotplot showing REACTOME terms found for the HaCaT cell line, with a maximum of 10 terms per category. Top terms are selected based on false discovery rate (FDR) and the odds ratio (OR). The *x* axis shows the cell cycle phases. Term names are shown on the y axis. The size of each dot is proportional to the OR of the associated term on the *y* axis. The colour scale is created using a -log10 transformation of the FDR which was calculated using the Benjamini-Hochberg approach.

**Figure 7.**
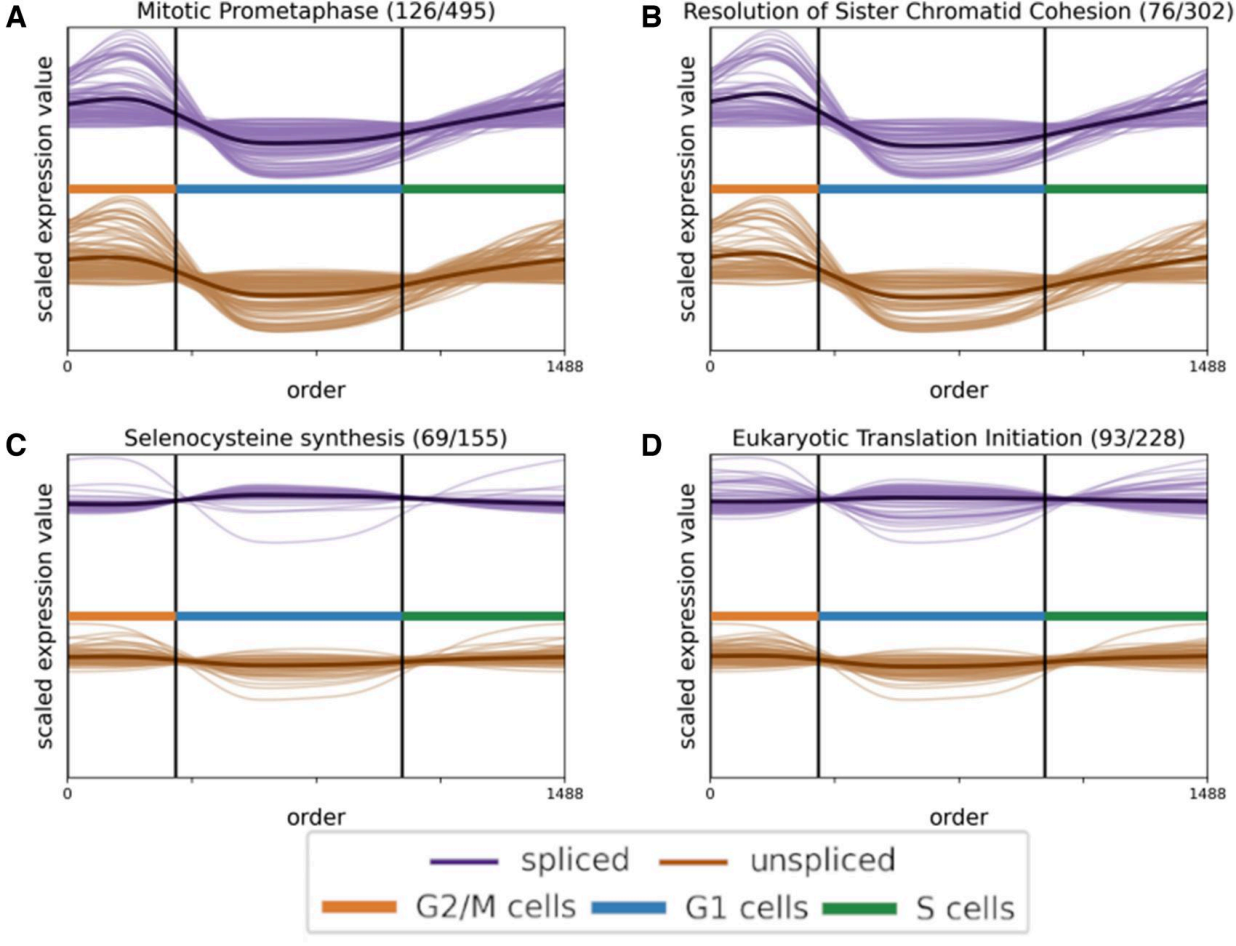
Line plots for the HaCaT cell line showing spliced and unspliced expression trajectories for genes from selected REACTOME pathways. Plots A and B highlight pathways peaking in G2/M—Mitotic Prometaphase and Resolution of Sister Chromatid Cohesion—while Plots C and D focus on G1 peaking pathways—Selenocysteine Synthesis and Eukaryotic Translation Initiation. Only genes with an adjusted p value < 0.01 are included, and each pathway’s mean spliced and unspliced trend is overlaid as a bold, dark line. Values are scaled, and plot titles list both the number of genes shown and the total genes in each REACTOME pathway.

To investigate the extent with which our methods and results generalized into other data, we ran our analysis pipeline on publicly available scRNA-seq data from two other cell lines: the human T lymphocyte cell line Jurkat and the human embryonic kidney cell line 293T. Quality control (Tables 7 and 8, available as supplementary data at *Bioinformatics Advances* online) and pseudotime assignment (Figs 6 and 7, available as supplementary data at *Bioinformatics Advances* online) showed that the four technical replicates for each cell line were comparable but slightly more variable than those from our HaCaT data. We identified 4334 and 4369 significant genes in the 293T and Jurkat data and these genes showed similar patterns in delays as the significant HaCaT genes (Fig. 8, available as supplementary data at *Bioinformatics Advances* online, File 1, available as supplementary data at *Bioinformatics Advances* online). With an odds ratio greater than one representing a higher likelihood of overlap of significant genes, we observe that general gene overlap, irrespective of cell cycle phase, shows a lower odd of obtaining significant gene overlap (Table 9, available as supplementary data at *Bioinformatics Advances* online). To further evaluate these results, we investigated to what extent genes with specific phase assignments overlapped between the cell lines. We used three possible ways of assigning genes to the three main pseudotime phases (G1, S, and G2/M), based on the locations of peak velocity, peak expression, and start of transcription (i.e. velocity > 0). Though variation is observed between the methods, the general trend is a odds ratio greater than one along with a significant *P* value for genes associated with the S and G2/M phases. Overall, phase assignment based on expression appeared to be the better method, likely due to the use of gene expression for the establishment of the cell cycle pseudotime, as opposed to gene velocity.

The altering direction of the odds ratio when comparing the general overlap with cell cycle phase overlap points towards some variability within the data. To accommodate this, we implement the use of a delay threshold whose identification is described in the previous section. We again compute the gene and phase overlap, but this time limiting the phase overlap to the peak expression category (Table 10, available as supplementary data at *Bioinformatics Advances* online). These results show some improvement of significance in the phase assignment, however there are still indications of strong variability within the data. This is further explored in the section titled: Filtering for house-keeping genes.

### 3.8 Applying the Velocyto model

For comparisons, we applied the Velocyto model to the scRNA-seq data from the three cell lines (Fig. 5; Figs 6 and 7, available as supplementary data at *Bioinformatics Advances* online). We obtained velocity fields which clearly show the different cell cycle phases along with their predicted next phase. Velocyto was able to accurately predict the transition from S phase to G2/M phase and the transition from G2/M to G1. The G1 to S transition is not predicted by Velocyto as it is not exclusively dependant on gene expression. It is instead thought to largely depend on critical cell size (Barberis *et al.* 2007) and therefore cannot be visualized using this method. However, we note that the overall trend, represented by the continuous black line in Fig. 5, shows a trajectory from G1 to G2/M. Velocyto also predicted the same overall trend in all four Jurkat and 293T replicates (Figs 6 and 7, available as supplementary data at *Bioinformatics Advances* online). Additionally, we check the results obtained from scVelo (Fig. 11, available as supplementary data at *Bioinformatics Advances* online), which show differing results when compared to Velocyto, where the velocity stream and latent time end somewhere between the S and G2M clusters. The divergence between Velocyto and scVelo show that neither is ideal for the creation of a cell cycle pseudotime, however the Velocyto results show a behaviour than can be explained through biology while the same cannot be said with scVelo where the terminal state is situated roughly between S and G2M.

### 3.9 Pathway enrichment for pseudotime validation

To biologically validate our findings, we performed an over-representation analysis using gprofiler (Raudvere *et al.* 2019). Considering three sets of genes assigned to the three cell cycle phases, we expect the sets to be enriched for functions related to their respective phases; i.e. we expect S phase genes to be enriched for functions related to DNA replication and DNA repair. Indeed, when analysing the significant HaCaT genes phase assigned based on peak expression, both S and G2/M phase genes were enriched for expected biological pathways (Fig. 6).

For S phase, the most significant terms were related to DNA repair, which is consistent with DNA repair having a critical role during DNA replication (Hustedt and Durocher 2016). For G2/M, we primarily observed mitosis-related terms. Genes in G1 were primarily defined by translational initiation and preparation of ribosomal subunits. Although G1 phase is defined by cellular growth driven by protein production, these pathways are broad in nature and may be associated with general house-keeping functions rather than G1 specifically (see the next section). Running the same over-representation analysis on the Jurkat, 293T and HeLa results, gave strikingly similar results across the different cell lines (Fig. 12, available as supplementary data at *Bioinformatics Advances* online). Noticeably, in both the 293T, Jurkat and HeLa cell lines, S phase associated pathways were also found within G1, but at a reduced significance. This indicates that the assigned G1-S boundary in these two datasets may be slightly shifted in favour of G1. We initially suspected that the number of cell cycle genes found would be the cause of this, however, the differences between cell lines are small (Table 11, available as supplementary data at *Bioinformatics Advances* online). An additional possibility is that there are alternate sources of variability present within the data thus affecting the phase assignment as indicated by both the PCAs (Figs 6 and 7, available as supplementary data at *Bioinformatics Advances* online) and the velocity fields (Figs 9 and 10, available as supplementary data at *Bioinformatics Advances* online). An over-representation analysis of the genes which did not pass the *t*-test filter (Fig. 13, available as supplementary data at *Bioinformatics Advances* online) found no significant REACTOME cell-cycle terms, suggesting that the *t*-test filter retained the cell-cycle related genes.

### 3.10 Verifying the G1 phase results

To investigate the broad REACTOME pathways found to be associated with the G1 phase, we plotted the gene trajectories of selected REACTOME pathways associated with the G1 or G2/M phases (Fig. 7). Whereas both the genes associated with G1 and G2/M phase pathways mainly peaked in their respective phases, the genes in the G2/M phase pathways generally had much higher peak expression than the genes in the G1 pathways. Specifically, the expression trajectories for the G1 phase pathway genes seemed to be consistent with constant gene activity and a very mild increase in G1. Such a low increase could potentially be explained by large fluctuations in cell cycle genes expressed in S and G2/M phases causing small but consistent inverse fluctuations in the relative expression profiles of house-keeping genes rather than cell cycle-dependent expression.

### 3.11 Filtering for house-keeping genes

To identify and remove genes with house-keeping behaviour, we implemented a gene filtering method based on gene expression throughout the cell cycle phases, removing genes having a cell cycle variability below a certain threshold. To determine a basis for the threshold, we calculated the mean variability of the genes in the two G2/M and G1 REACTOME pathways in Fig. 7. The variability was calculated based on the log10 transformed gene expression (Table 12, available as supplementary data at *Bioinformatics Advances* online) in order to account for the heteroskedasticity of count data as well as different genes having different average expression levels potentially due to their varying functions.

Based on the expression variation for the putative housekeeping genes compared with the G2/M phase genes, we decided to filter out genes based on the log10 cell cycle variance, where the median log10 variance for each pathway is calculated, and the mean of the median is selected as the variance threshold. Hence this creates a variance threshold that will be specific for each cell line. Following this filter (in addition to the *t*-test and delay filters), 1140, 2213, and 1983 significant genes remained for HaCaT, 293T, and Jurkat respectively. Subsequent pathway enrichment analysis of the remaining genes showed a marked decrease in the significance of the remaining G1 genes (Fig. 14, available as supplementary data at *Bioinformatics Advances* online). All G1 pathways were no longer considered significant and therefore do not appear in the plot. Furthermore, the G1 genes passing the variation filter showed marked increased expression during the G1 phase (Fig. 15, available as supplementary data at *Bioinformatics Advances* online). We found similar results in the other three cell lines (Fig. 16, available as supplementary data at *Bioinformatics Advances* online), with the exception that certain G1 pathways remain, however at a reduced significance. We also perform a chi-square analysis for gene overlap, with this variance filter, in addition to the previous measures of identifying significant genes (*t*-test and negative delay threshold) (Table 13, available as supplementary data at *Bioinformatics Advances* online) In this table we observe several significant odd ratios above one, indicating a higher likelihood of overlap, these odds are increased from the previous gene overlap analysis which used the *t*-test and delay thresholding alone (Table 10, available as supplementary data at *Bioinformatics Advances* online). Put together, these findings confirmed that filtering genes with low variation (below the mean of the median) effectively removed genes whose apparent periodic expression could be experimental artefacts.

### 3.12 Cross-reference significant cell cycle genes with TCGA

Genes with cell-cycle dependent expression tend to be upregulated in dividing cells. To further validate the significant genes found using the *t*-test, delay threshold, and cell cycle variance threshold, we sought to compare them with upregulated genes within The Cancer Genome Atlas (TCGA). We, therefore, identified genes (826) up-regulated in all registered cancers compared with the corresponding normal tissues in the TCGA database (Giordano 2014). Our assumption was that these genes will primarily be cell cycle genes upregulated in cancers in general and should therefore be found in our list of identified significant genes.

When comparing the TCGA genes with the filtered genes from our cell cycle analyses, all three cell lines had significant overlap with the TCGA genes (Tables S14 and 15, available as supplementary data at *Bioinformatics Advances* online). The overall cell cycle phase trends were also similar, with G1 genes having similar to or significantly less overlap than expected and S and G2/M having significantly more overlapping genes than expected. This trend was even stronger when only considering common significant genes between the three cell lines. These results indicate that genes found to be significant in both G2/M and S phase are relevant for cancer proliferation, whereas most or all genes found to be associated with the G1 phase are not.

## 4 Conclusion

We have shown that our scRNA-seq protocol creates reproducible results by comparing all results with its technical replicate counter part. We also demonstrated the protocol’s ability to sort cells along a cell cycle based pseudotime order while using the S-G2/M transition point as the start of the pseudotime. Additionally, we validated the method that calculates and uses gene velocity to identify genes of interest. These genes are considered to be significant by a *t*-test based on the presence of positive and negative gene velocity. The list of relevant genes is further filtered via the removal of genes with significant negative cell cycle delays and house-keeping behaviour. Using our filtered significant genes we found significant overlap with genes up-regulated in cancers from the TCGA database.

Our method for creating a pseudotime ordering operates on the assumption that a gene list of 172 genes is representative of the cell cycle and that the expression patterns of these genes are mainly related to the cell cycle. Although this may be a limitation in certain cases, it may double as an advantage. This assumption allows this method to be easily transferable to various biological pathways provided that they contain an adequate number of representative genes.

A second limitation of this study is the case of variability within scRNA-seq data. We have demonstrated that variability within technical replicates exists and that we can adjust for this variability by merging technical replicates. However, not all studies may have technical replicates available. This aspect should be considered when employing this method with single replicate studies.

Overall, we show an efficient way of studying the cell cycle and its various elements all while circumventing the need to perform cell cycle synchronization or selection experiments.

## Supplementary Material

vbaf123_Supplementary_Data

## Data Availability

The code used can be found at our github (https://github.com/Ylefol/CC_vel), or via Zenodo (DOI: 10.5281/Zenodo.10477761). Loom files for the HaCaT replicates are available on our github, the loom files for the 293T, Jurkat, and HeLa can be provided upon request. Raw sequences for the HaCaT replicates can be found at ENA (https://www.ebi.ac.uk/) using the PRJEB71981 accession number. The 293T and Jurkat raw data can be obtained from https://support.10xgenomics.com/single-cell-gene-expression/datasets/1.1.0. The raw data for HeLa can be obtained from https://www.ncbi.nlm.nih.gov/sra/? term=SRR10741798
