# Supplementary material for: Using pseudotime derivative on single-cell RNA sequencing data to identify genes undergoing cell cycle regulation
Source: Bioinform Adv. 2025 May 29;5(1):vbaf123. doi: 10.1093/bioadv/vbaf123 (PMC12255884; doi:10.1093/bioadv/vbaf123)
Supplement: vbaf123_Supplementary_Data [file vbaf123_supplementary_data.zip › Supplementary_tables_and_figures.pdf]

Using pseudotime derivative on single cell RNA sequencing data to identify genes undergoing cell cycle regulation

Yohan Lefol,[1,2,3,4*] Geir Amund Svan Hasle,[2,5], Siv Anita Hegre,[2] Helle Samdal,[2] Pål Sætrom,[2,5,6,7,*]

[1] Génie physiologique, biotechnologique et informatique, Université de Poitiers, Poitiers 8600, France
[2] Department of Clinical and Molecular Medicine, Norwegian University of SCience and Technology, NTNU, Trondheim 7030, Norway
[3] Department of Microbiology, Institue of Clinical Medicine, University of Oslo, N-0373 Oslo, Norway
[4] CRESCO, Center for Embryology and Healthy Development, University of Oslo, N-0373 Oslo, Norway
[5] St. Olavs hospital HF, Sentral Stab, NO-7006 Trondheim, Norway
[6] Department of Computer and Information Science, Norwegian University of Science and Technology, NTNU, Trondheim, 7491, Norway
[7] K.G. Jebsen Center for Genetic Epidemiology, Norwegian University of Science and Technology, NTNU, Trondheim 7491, Norway

* To whom correspondence should be addressed.
Tel: +47 465 02 489; Email: yohan.lefol@gmail.com or
Email: pål.satrom@ntnu.no

SUPPLEMENTARY DATA

Contents:

Supplementary File S1 (PDF)
Supplementary File S2 (excel)
Supplementary Tables S1-S15
Supplementary Figures S1-S16

Table S1
Quality control results showing the amount of cells and genes removed by each filter in the HaCaT replicates.

Category	Rep. A	Rep. B
Initial number of cells	2933	2948
Murine cells	227	526
Cells with less than 500 gene reads	50	52
Cells with less than 4000 total reads	77	98
Cells with more than 25% unspliced reads	11	18
Cells with less than 10% unspliced reads	15	18
Cells with mitochondrial reads	151	151
Final number of cells	2399	2085
Initial number of genes	60617	60617
Genes expressed in less than 5 cells	41900	42080
Genes with no unspliced reads	2536	2535
Final number of genes	16181	16002

Table S2

Gprofiler results for the differential genes of G1 cells vs S cells in the S phase section of the PCA plot in HaCat_A.

REAC		stats	
Term name	Term ID	p_{adj}	$-\log_{10}(p_{adj})$
Cell Cycle	REAC:R-HSA-16...	2.168×10^{-110}	
Cell Cycle, Mitotic	REAC:R-HSA-69...	1.766×10^{-88}	
Cell Cycle Checkpoints	REAC:R-HSA-69...	5.619×10^{-49}	
M Phase	REAC:R-HSA-68...	8.046×10^{-46}	
Mitotic Prometaphase	REAC:R-HSA-68...	6.202×10^{-44}	
Mitotic Metaphase and Anaphase	REAC:R-HSA-25...	1.556×10^{-36}	
Mitotic Anaphase	REAC:R-HSA-68...	7.577×10^{-36}	
S Phase	REAC:R-HSA-69...	4.804×10^{-33}	
DNA Repair	REAC:R-HSA-73...	4.575×10^{-31}	
Resolution of Sister Chromatid Cohesion	REAC:R-HSA-25...	1.178×10^{-28}	
Separation of Sister Chromatids	REAC:R-HSA-24...	1.005×10^{-27}	
Mitotic Spindle Checkpoint	REAC:R-HSA-69...	3.357×10^{-27}	
Synthesis of DNA	REAC:R-HSA-69...	3.791×10^{-26}	
Amplification of signal from the kinetochores	REAC:R-HSA-14...	1.264×10^{-25}	
Amplification of signal from unattached kinetochores ...	REAC:R-HSA-14...	1.264×10^{-25}	
Mitotic G1 phase and G1/S transition	REAC:R-HSA-45...	4.306×10^{-25}	
DNA strand elongation	REAC:R-HSA-69...	1.039×10^{-24}	
EML4 and NUDC in mitotic spindle formation	REAC:R-HSA-96...	1.207×10^{-24}	
DNA Double-Strand Break Repair	REAC:R-HSA-56...	1.477×10^{-24}	
Processing of Capped Intron-Containing Pre-mRNA	REAC:R-HSA-72...	1.991×10^{-24}	

Table S3
Gprofiler results for the differential genes of G1 cells vs G2M cells in the G2M phase section of the PCA plot in HaCat_A.

REAC		stats	
Term name	Term ID	p_{adj}	$-\log_{10}(p_{adj})$
Cell Cycle	REAC:R-HSA-16...	3.012×10^{-83}	
Cell Cycle, Mitotic	REAC:R-HSA-69...	3.426×10^{-73}	
M Phase	REAC:R-HSA-68...	4.822×10^{-48}	
Mitotic Prometaphase	REAC:R-HSA-68...	1.266×10^{-45}	
Mitotic Anaphase	REAC:R-HSA-68...	8.189×10^{-45}	
Cell Cycle Checkpoints	REAC:R-HSA-69...	8.852×10^{-45}	
Mitotic Metaphase and Anaphase	REAC:R-HSA-25...	1.140×10^{-44}	
Resolution of Sister Chromatid Cohesion	REAC:R-HSA-25...	5.404×10^{-39}	
Separation of Sister Chromatids	REAC:R-HSA-24...	1.106×10^{-37}	
Mitotic Spindle Checkpoint	REAC:R-HSA-69...	3.195×10^{-35}	
EML4 and NUDC in mitotic spindle formation	REAC:R-HSA-96...	1.173×10^{-32}	
Amplification of signal from unattached kinetochores ...	REAC:R-HSA-14...	3.606×10^{-32}	
Amplification of signal from the kinetochores	REAC:R-HSA-14...	3.606×10^{-32}	
RHO GTPases Activate Formins	REAC:R-HSA-56...	2.081×10^{-31}	
RHO GTPase Effectors	REAC:R-HSA-19...	9.692×10^{-21}	
Signaling by Rho GTPases, Miro GTPases and RHOBT...	REAC:R-HSA-97...	2.673×10^{-15}	
Signaling by Rho GTPases	REAC:R-HSA-19...	3.151×10^{-15}	
Chromosome Maintenance	REAC:R-HSA-73...	9.624×10^{-15}	
G2/M Transition	REAC:R-HSA-69...	1.388×10^{-13}	
S Phase	REAC:R-HSA-69...	1.564×10^{-13}	

Bar scale: 0 to ≥16

Table S4
Gprofiler results for the HaCaT genes with significant negative delays.

REAC Term name	Term ID	stats p_{adj}	$-\log_{10}(p_{adj})$
Formation of a pool of free 40S subunits	REAC:R-HSA-72...	5.116×10^{-3}	
L13a-mediated translational silencing of Ceruloplasmi...	REAC:R-HSA-15...	1.017×10^{-2}	
GTP hydrolysis and joining of the 60S ribosomal subunit	REAC:R-HSA-72...	1.085×10^{-2}	
Eukaryotic Translation Initiation	REAC:R-HSA-72...	1.676×10^{-2}	
Cap-dependent Translation Initiation	REAC:R-HSA-72...	1.676×10^{-2}	
Viral mRNA Translation	REAC:R-HSA-19...	1.953×10^{-2}	
Peptide chain elongation	REAC:R-HSA-15...	1.953×10^{-2}	
rRNA processing	REAC:R-HSA-72...	2.318×10^{-2}	
Eukaryotic Translation Termination	REAC:R-HSA-72...	2.585×10^{-2}	
Eukaryotic Translation Elongation	REAC:R-HSA-15...	2.585×10^{-2}	
Selenocysteine synthesis	REAC:R-HSA-24...	2.585×10^{-2}	
Nonsense Mediated Decay (NMD) independent of the ...	REAC:R-HSA-97...	2.959×10^{-2}	
Response of EIF2AK4 (GCN2) to amino acid deficiency	REAC:R-HSA-96...	4.354×10^{-2}	
Respiratory electron transport	REAC:R-HSA-61...	4.631×10^{-2}	

Table S5
Gprofiler results for the 293T genes with significant negative delays.

REAC Term name	Term ID	stats p_{adj}	$-\log_{10}(p_{adj})$
Signaling by Rho GTPases, Miro GTPases and RHOBT...	REAC:R-HSA-97...	8.072×10^{-4}	
RHO GTPase cycle	REAC:R-HSA-90...	1.240×10^{-3}	
RHOC GTPase cycle	REAC:R-HSA-90...	1.300×10^{-3}	
Signaling by Rho GTPases	REAC:R-HSA-19...	1.493×10^{-3}	
RHOB GTPase cycle	REAC:R-HSA-90...	2.998×10^{-3}	
SUMOylation	REAC:R-HSA-29...	8.263×10^{-3}	
Cell Cycle	REAC:R-HSA-16...	1.213×10^{-2}	
SUMO E3 ligases SUMOylate target proteins	REAC:R-HSA-31...	1.347×10^{-2}	
Cell Cycle, Mitotic	REAC:R-HSA-69...	3.285×10^{-2}	

Table S6

Gprofiler results for the Jurkat genes with significant negative delays.

REAC Term name	Term ID	stats p_{adj}	$-\log_{10}(p_{adj})$
Metabolism of RNA	REAC:R-HSA-89...	7.172×10^{-6}	
tRNA processing	REAC:R-HSA-72...	7.688×10^{-4}	
Influenza Viral RNA Transcription and Replication	REAC:R-HSA-16...	1.841×10^{-2}	
Influenza Infection	REAC:R-HSA-16...	2.392×10^{-2}	
tRNA processing in the nucleus	REAC:R-HSA-67...	3.891×10^{-2}	
Host Interactions of HIV factors	REAC:R-HSA-16...	3.938×10^{-2}	
Nuclear Pore Complex (NPC) Disassembly	REAC:R-HSA-33...	4.491×10^{-2}	

Table S7

Quality control results showing the amount of cells and genes removed by each filter in the 293T replicates.

Category	Replicate A	Replicate B	Replicate C	Replicate D
Initial number of cells	2899	2898	2897	2898
Murine cells	0	0	0	0
Cells with less than 500 gene reads	0	0	0	0
Cells with less than 4000 total reads	224	114	186	186
Cells with more than 25% unspliced reads	50	66	45	48
Cells with less than 10% unspliced reads	20	24	13	21
Cells with more than 0% of mitochondrial reads	0	0	0	0
Final number of cells	2605	2694	2653	2643
Initial number of genes	60623	60623	60623	60623
Genes expressed in less than 5 cells	43849	43354	43731	43716
Genes with no unspliced reads	2438	2405	2446	2400
Final number of genes	14336	14864	14446	14507

Table S8

Quality control results showing the amount of cells and genes removed by each filter in the jurkat replicates.

Category	Replicate A	Replicate B	Replicate C	Replicate D
Initial number of cells	3261	3259	3261	3261
Murine cells	0	0	0	0
Cells with less than 500 gene reads	1	1	1	1
Cells with less than 4000 total reads	256	187	259	213
Cells with more than 25% unspliced reads	62	81	66	76
Cells with less than 10% unspliced reads	1	2	3	3
Cells with more than 0% of mitochondrial reads	5	6	6	9
Final number of cells	2936	2982	2926	2959
Initial number of genes	60623	60623	60623	60623
Genes expressed in less than 5 cells	44649	44334	44672	44425
Genes with no unspliced reads	2197	2223	2182	2226
Final number of genes	13777	14066	13769	13972

Table S9

Odds ratio and associated P value results for the chi square test measuring gene overlap for all significant genes found within each cell line (Gene overlap) as well as genes associated to specific cell cycle phases which were identified using three different categories based on active transcription. The odds ratio appears first, with the pvalue following the pipe (|), significant p values are shown in bolded text.

Cell lines	Gene overlap	Phase	Phase peak velocity	Phase peak expression	Phase velocity start
HaCaT vs Jurkat	0.82 | **2.10**e−**04**	G1	0.67 | **1.72**e−**05**	0.69 | **6.49**e−**06**	1.53 | **1.39**e−**07**
		S	1.35 | **1.24**e−**06**	1.47 | **0.01**	0 | 0.88
		G2/M	1.20 | **0.03**	1.70 | **5.31**e−**22**	0.71 | **6.18**e−**11**
HaCaT vs 293T	0.89 | **0.03**	G1	0.96 | 0.78	0.91 | 0.24	1.42 | **2.99**e−**05**
		S	1.49 | **3.14**e−**13**	1.50 | 0.07	0 | 1
		G2/M	1.09 | 0.32	1.55 | **7.88**e−**16**	0.67 | **5.83**e−**14**
Jurkat vs 293T	0.82 | **3.30**e−**03**	G1	1.51 | **1.52**e−**04**	1.60 | **2.74**e−**18**	1.68 | **6.99**e−**08**
		S	2.13 | **2.76**e−**33**	8.26 | **1.18**e−**18**	0 | 1
		G2/M	1.18 | **3.88**e−**03**	2.09 | **2.20**e−**37**	0.84 | **5.87**e−**03**

Table S10
Chi-square gene overlap results using genes which are significant based on an adjusted pvalue < 0.01 from a t-test as well as genes which pass a cell cycle delay threshold derived from the 95th percentile of the lower tail of a normally distributed curve of delay values. The contingency table is shown first, followed by the odds ratio and the associated p-value (in bold if significant). The odds ratio and p-value are separrated by a pipe (|). Phase overlap is also performed, where the test checks if different cell lines map the same gene to the same phase. Only significant genes have been used to measure phase overlap.

<table>
<tr><th>Cell lines</th><th>Cont-table</th><th>Gene overlap</th><th>Phase</th><th>Cont-table</th><th>Phase peak expression</th></tr>
<tr><td rowspan="3">HaCaT vs Jurkat</td><td rowspan="3">2327 1445
2146 1293</td><td rowspan="3">0.97 | 0.55</td><td>G1</td><td>235 2163
474 3046</td><td>0.70 | 2.41e−05</td></tr>
<tr><td>S</td><td>76 93
2028 3721</td><td>1.50 | 0.01</td></tr>
<tr><td>G2/M</td><td>890 937
1422 2669</td><td>1.78 | 3.84e−24</td></tr>
<tr><td rowspan="3">HaCaT vs 293T</td><td rowspan="3">2300 1375
2173 1322</td><td rowspan="3">1.02 | 0.74</td><td>G1</td><td>273 2089
439 3047</td><td>0.91 | 0.25</td></tr>
<tr><td>S</td><td>40 48
2061 3699</td><td>1.49 | 0.08</td></tr>
<tr><td>G2/M</td><td>919 1017
1401 2511</td><td>1.62 | 1.27e−17</td></tr>
<tr><td rowspan="3">Jurkat vs 293T</td><td rowspan="3">2354 1418
1321 885</td><td rowspan="3">1.11 | 0.06</td><td>G1</td><td>1276 1130
1053 1634</td><td>1.75 | 5.41e−23</td></tr>
<tr><td>S</td><td>18 151
71 4853</td><td>8.15 | 3.79e−18</td></tr>
<tr><td>G2/M</td><td>909 922
1036 2226</td><td>2.12 | 2.85e−36</td></tr>
</table>

Table S11
Table of known cell cycle genes for each phase found in each replicate of each cell line used.

	HaCaT		293T				Jurkat			
Replicates	A	B	A	B	C	D	A	B	C	D
G1 - 8 known genes	8	8	7	7	7	8	8	8	8	8
S - 74 known genes	74	73	73	72	72	73	74	74	73	74
G2/M - 90 known genes	90	89	89	89	90	89	89	90	88	90

Table S12
Gene expression variance of target REACTOME pathways throughout the phases of the cell cycle. The first column indicates the REACTOME pathway, the second shows the variance while not transforming the expression data, the third column shows the found variance while having log10 transformed the gene expression data.

REACTOME pathway	Variance	log10.Var
Mitotic Prometaphase	0.183	0.021
Resolution of Sister Chromatid Cohesion	0.195	0.031
Selenocysteine synthesis	61.775	2.70e−04
Eukaryotic Translation Initiation	34.598	4.81e−05

Table S13
Chi-square gene overlap results using genes which are significant based on an adjusted pvalue < 0.01 from a t-test, a cell cycle variance filter as well as genes which pass a cell cycle delay threshold derived from the 95th percentile of the lower tail of a normally distributed curve of delay values. The contingency table is shown first, followed by the odds ratio and the associated p-value (in bold if significant). The odds ratio and p-value are separrated by a pipe (|). Phase overlap is also performed, where the test checks if different cell lines map the same gene to the same phase. Only significant genes have been used to measure phase overlap.

Cell lines	Cont-table	Gene overlap	Phase	Cont-table	Phase peak expression
HaCaT vs Jurkat	441, 1542<br>700, 4528	1.85 | **5.28e−20**	G1	98, 1000<br>210, 1375	0.64 | **6.91e−04**
			S	63, 61<br>789, 1770	2.32 | **4.94e−06**
			G2/M	579, 523<br>495, 1086	2.43 | **3.72e−28**
HaCaT vs 293T	399, 1841<br>742, 4215	1.25 | **1.20e−03**	G1	104, 1068<br>204, 1579	0.75 | **0.03**
			S	30, 28<br>891, 2006	2.41 | **1.07e−03**
			G2/M	688, 675<br>583, 1009	1.76 | **4.46e−14**
Jurkat vs 293T	806, 1177<br>1407, 2588	1.26 | **4.85e−05**	G1	736, 699<br>672, 1283	2.01 | **7.59e−23**
			S	17, 107<br>43, 3223	11.91 | **3.20e−23**
			G2/M	795, 600<br>702, 1293	2.44 | **4.28e−36**

Table S14
The odds ratio between significant genes of each cell line with the significant upregulated genes found in the full TCGA RNAseq database (as of writing this manuscript). Genes were selected based on significance with a t-test (an adjusted pvalue below 0.01), a cell cycle delay threshold, and a cell cycle variance (the mean of the median variance between replicates of a cell line). Genes are split into cell cycle phases based on which phase they were found to be most highly expressed. P value of the chi-sqare results is also given, with significant p values being bolded. The 'intersect' row represents a list of genes found in all three cell lines, the phase boundaries used were from the HaCaT cell line.

Cell lines	Phase	Odds ratio	pvalue
HaCaT	G1	0.47	0.13
	S	4.22	**1.17e−22**
	G2/M	6.68	**1.94e−49**
293T	G1	0.76	0.13
	S	5.62	**1.44e−08**
	G2/M	0.72	0.05
Jurkat	G1	0.62	**0.01**
	S	4.32	**2.99e−09**
	G2/M	2.86	**9.76e−15**
Intersection	G1	0	0.75
	S	8.42	**2.02e−24**
	G2/M	10.36	**3.93e−42**

Table S15

Table containing contigency tables as well as observed and expected overlap for the chi square test performed between significant genes of each cell line with the significant upregulated genes found in the full TCGA RNAseq database Contingency tables for the various chi square test for gene overlap. Contingency tables are comparisons between significant genes of a cell line and the TCGA gene list. The top left slot contains significant genes present in both the cell line and TCGA list. Top right shows genes which are significant in the cell line but not the TCGA list, bottom left is the opposite. Bottom right shows genes which are non-significant in both cell lines.

Cell lines	Phase	Contingency table		Observed overlap	Expected overlap
HaCaT	G1	5	180	5	10.11
		219	3693		
	S	67	356	67	23.12
		157	3517		
	G2/M	102	431	102	29.14
		122	3442		
293T	G1	42	903	42	51.67
		182	2970		
	S	13	42	13	3.01
		211	3831		
	G2/M	53	1160	53	66.32
		171	2713		
Jurkat	G1	34	865	34	49.15
		190	3008		
	S	20	86	20	5.79
		204	3787		
	G2/M	102	876	102	53.47
		122	2997		
Intersection	G1	0	14	0	0.76
		224	3859		
	S	27	62	27	4.86
		197	3811		
	G2/M	41	82	41	6.72
		183	3791		

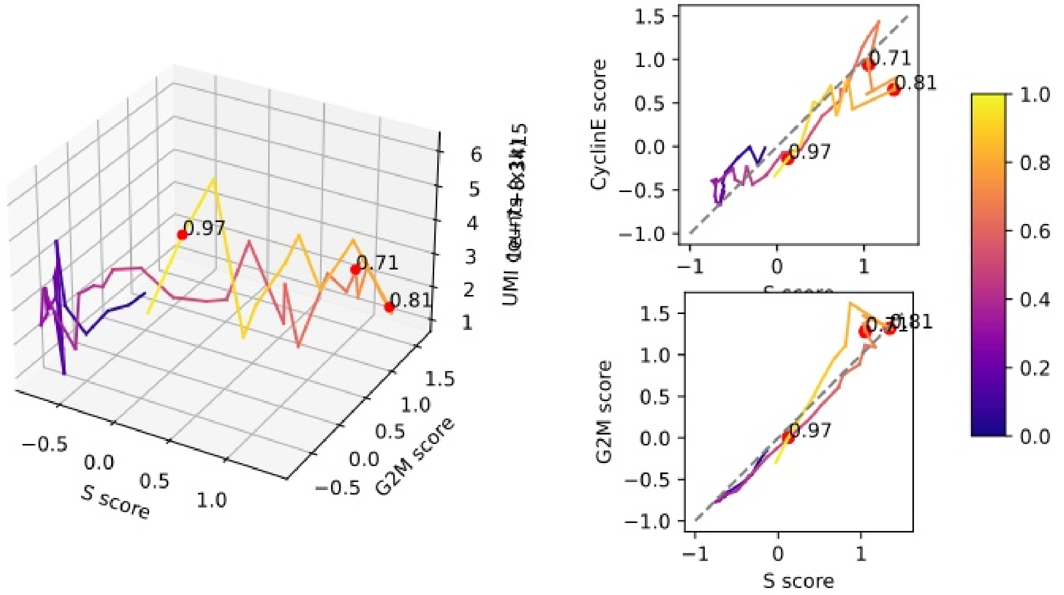

Figure S1
Figure resulting from the DeepCycle pipeline which shows the identified cell cycle transition points for HaCaT A based on the calculated theta score. The G1/S transition is marked by 0.71 (top CyclinE score), S/G2 is marked by 0.81 (where G2M score becomes greater than S), and M/G1 is marked by 0.97 (where the first bin where the number of RNA counts per cell drops). The color gradient represents the theta score.

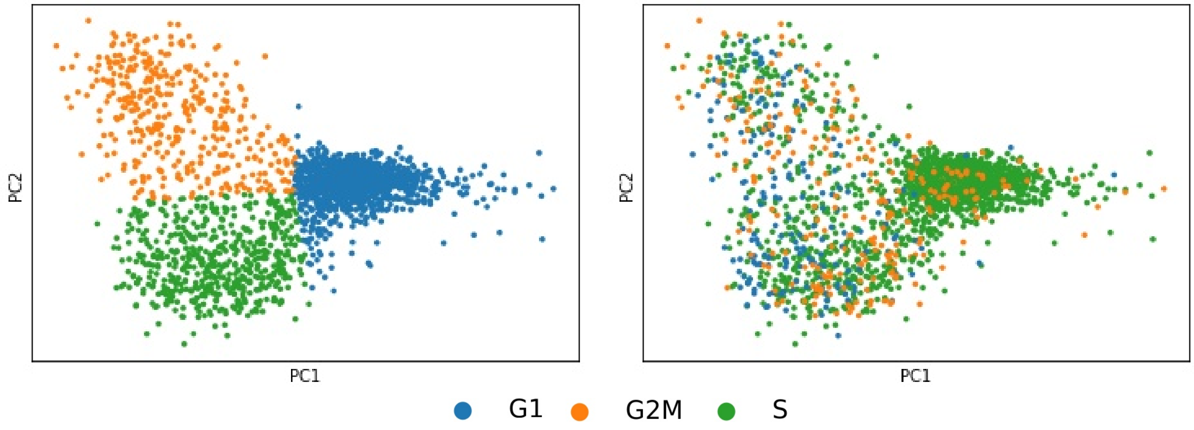

Figure S2
Two PCA plots showing phase association for the HaCaT A replicate where the left hand side shows our phase association method while the right hand side shows the DeepCycle phase association method.

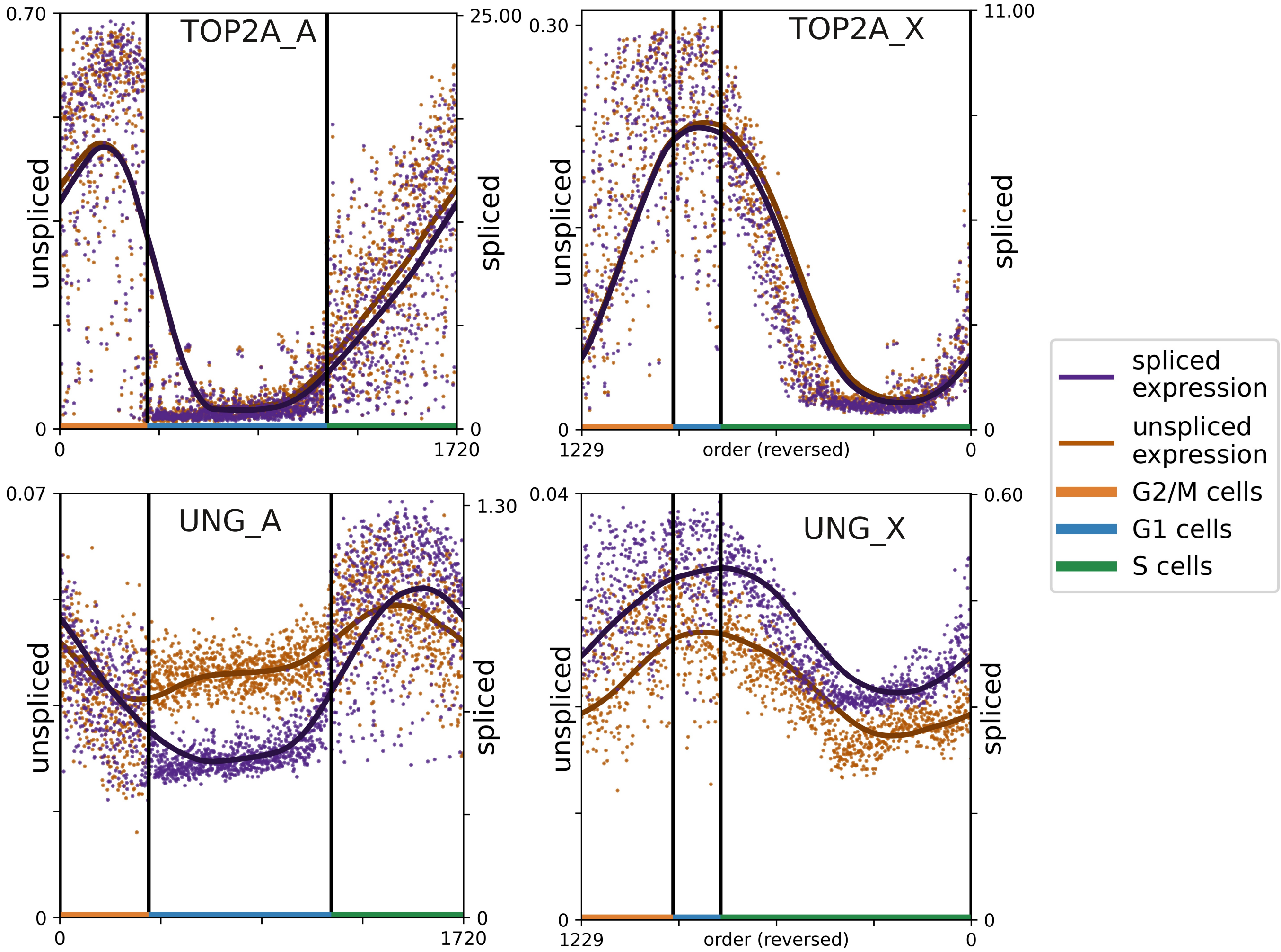

Figure S3

Gene plots for TOP2A and UNG where dots represent a singular cell and the lines represent the mean values. All plots utilize HaCaT A values, plots marked with '_A' (left hand side) show our method of phase assignement while plots with '_X' (right hand side) show the DeepCycle phase assignement method. Phase assignement is shown at the bottom of each plot with blue being G1, S being green, and G2M as orange. Each phase transistion is marked by vertical black lines.

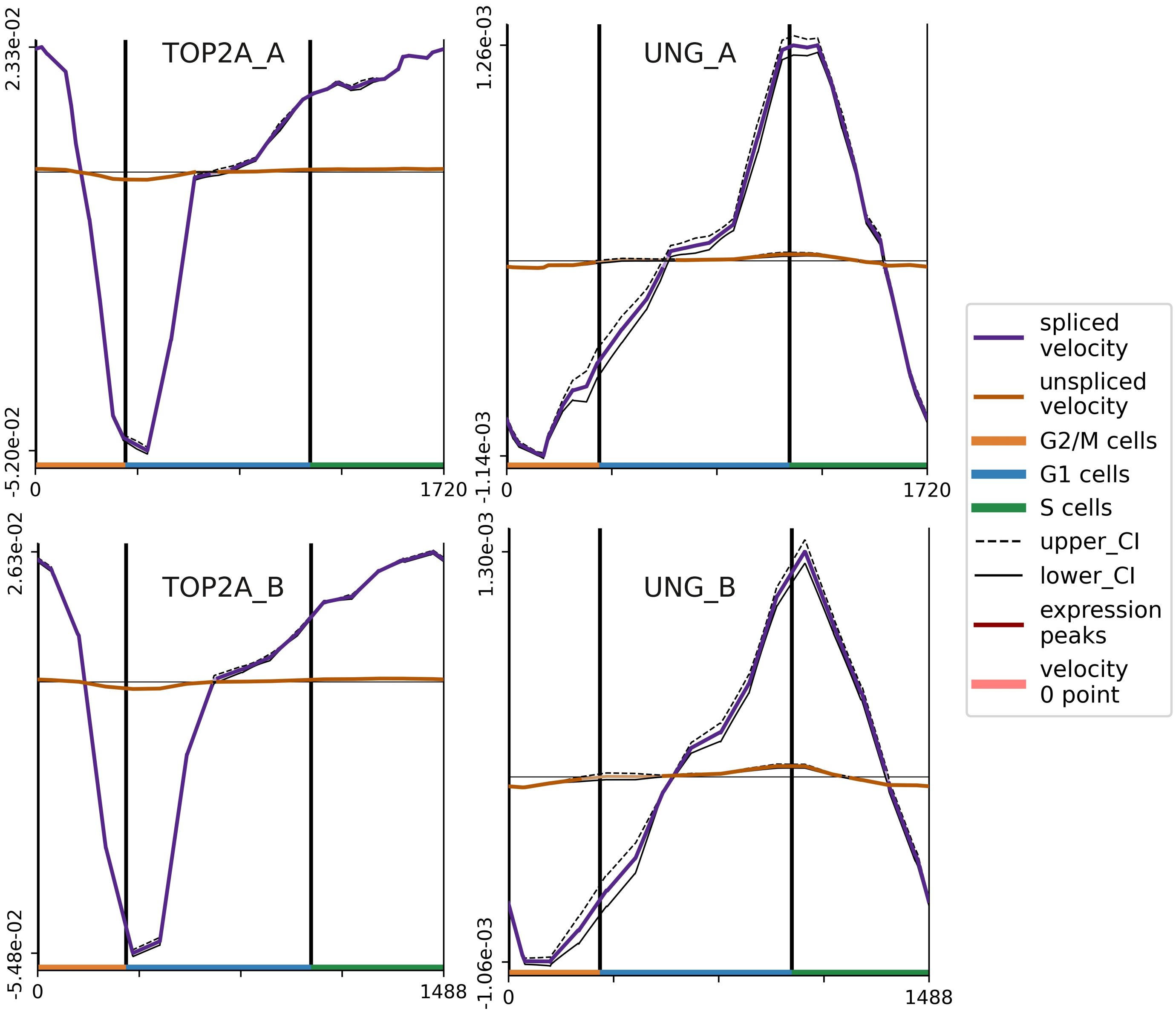

Figure S4
Velocity plots for HaCaT replicates of TOP2A (left side) and UNG (right side). Replicates are shown following the underscore after the gene name. Purple lines represent spliced expression, orange represents unspliced. The colored bars at the bottom represent cell cycle phases, with orange being G2M, blue G1, and green for S. Upper confidence intervals are shown as dotted black lines and lower confidence interval as a solid black line.

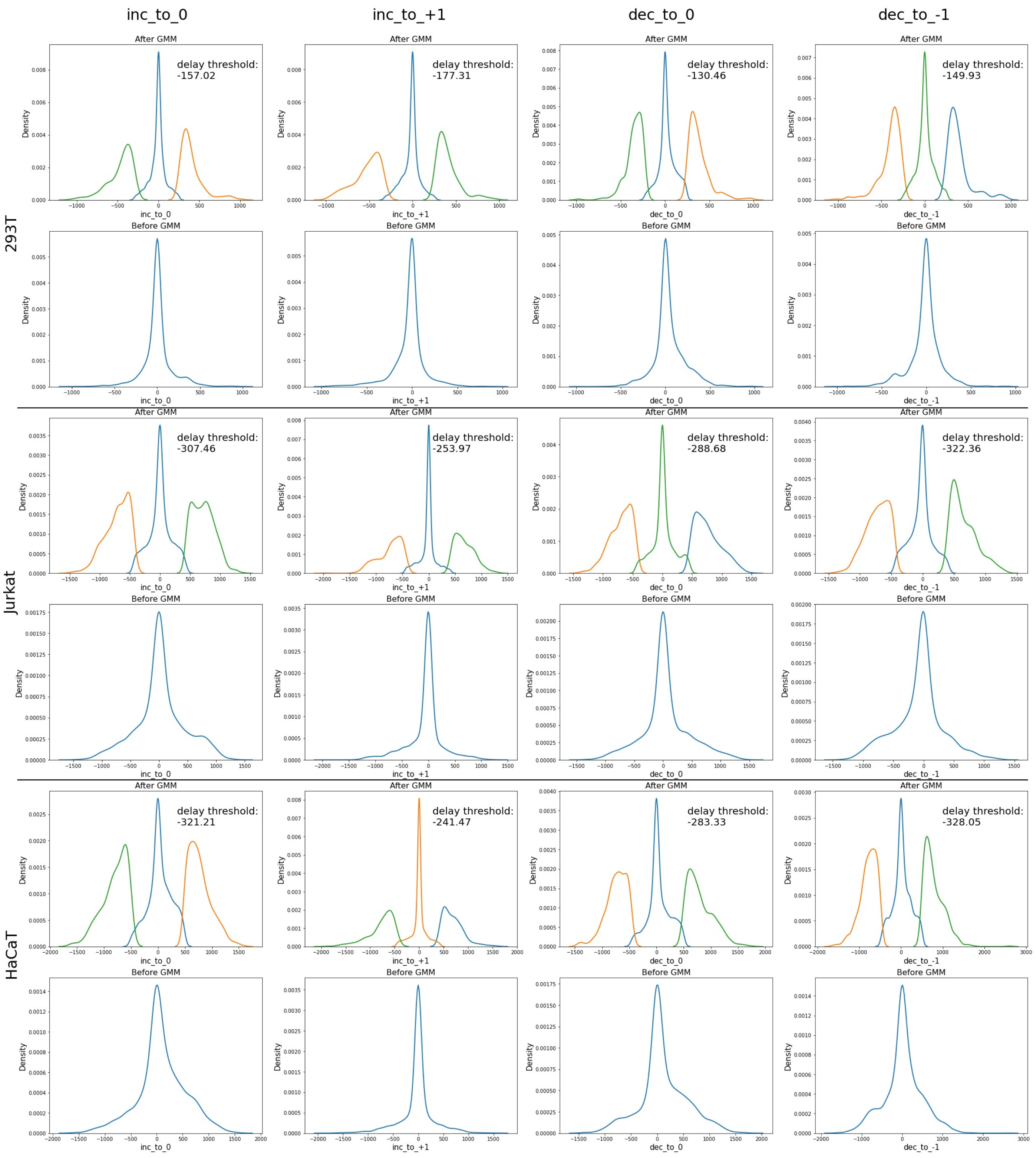

Figure S5

Kernel density plots showing cell delays per cell lines (rows) before and after the application of a Gaussian Mixture modelling. This is performed for all four delay categories measured (columns). The identified delay threshold is shown in the top right of each subplot.

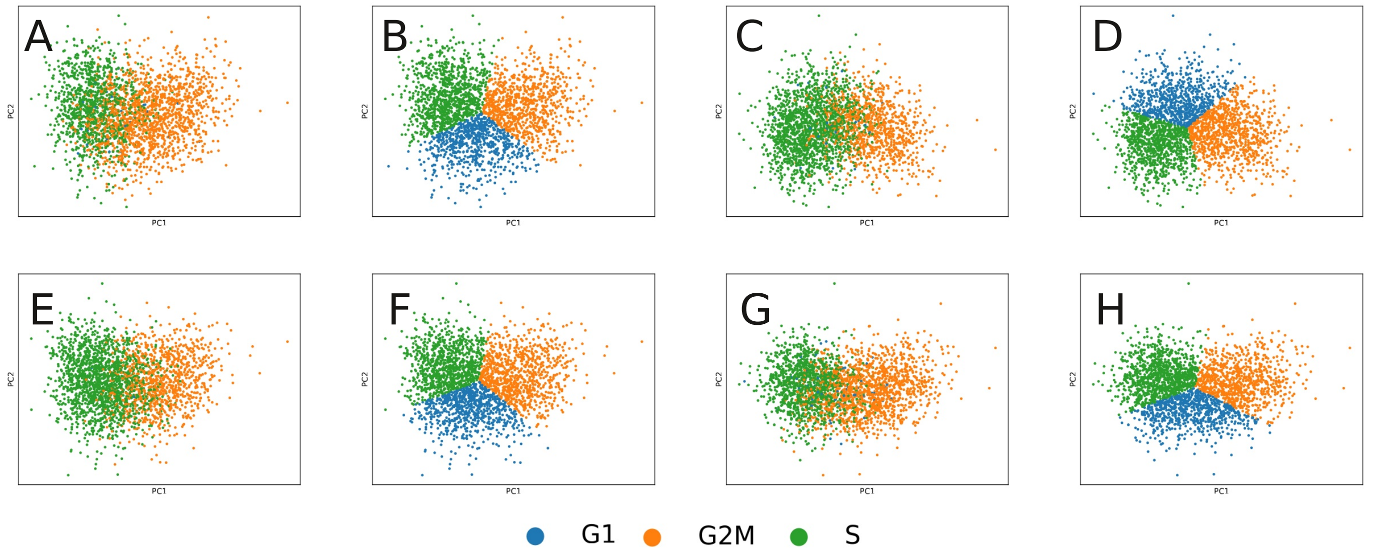

Figure S6

PCA plots representing the process of phase reassignment for all four replicates of the 293T cell line. PCAs with phase distribution were plotted after having regressed out n_counts(counts per cell before normalization) (a,c,g,f). PCAs were plotted again after establishing phase boundaries and performed phase reassignment according to those boundaries (b,d,f,g). Each point represents a cell, G1 cells are blue, S cells are green, and G2/M cells are orange.

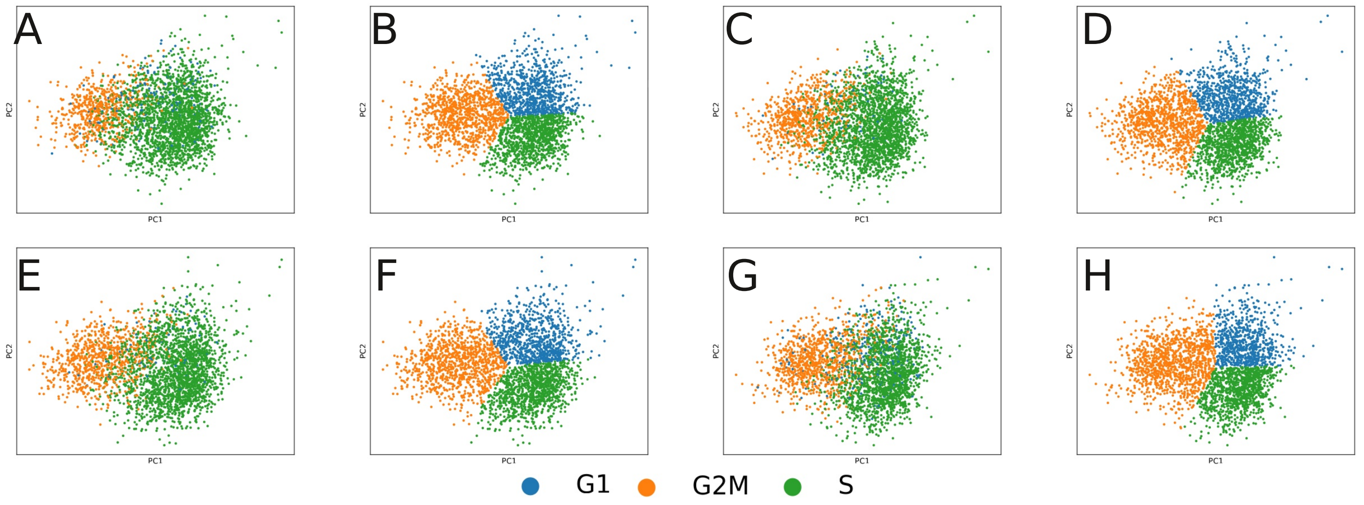

Figure S7

PCA plots representing the process of phase reassignment for all four replicates of the jurkat cell line. PCAs with phase distribution were plotted after having regressed out n_counts(counts per cell before normalization) (a,c,g,f). PCAs were plotted again after establishing phase boundaries and performed phase reassignment according to those boundaries (b,d,f,g). Each point represents a cell, G1 cells are blue, S cells are green, and G2/M cells are orange.

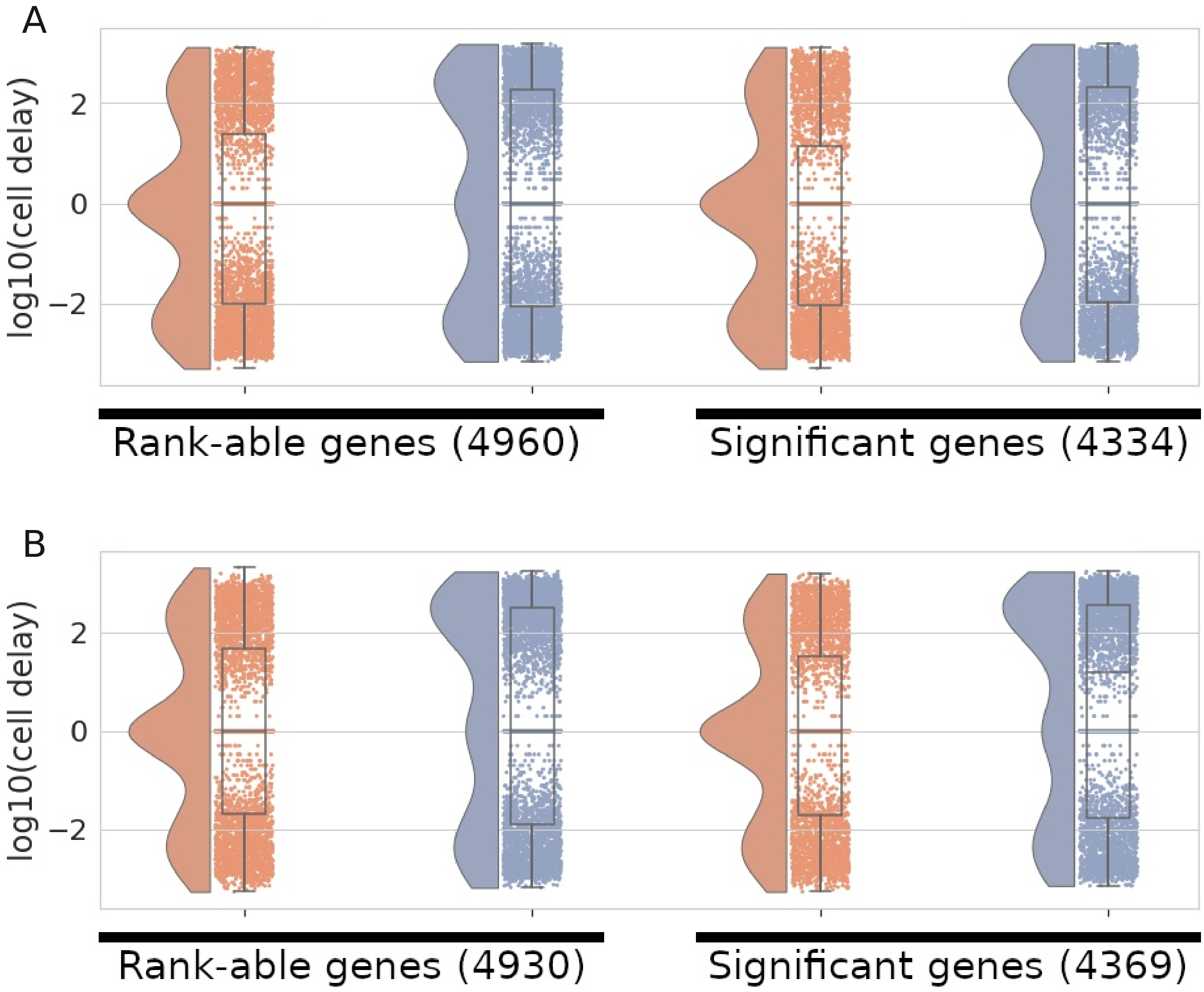

Figure S8
Raincloud plot for 293T(a) and jurkat(b) showing two delay categories using two different sets of genes. Orange shows the `increase to 1' and blue shows `decrease to 0'. Significant genes are genes with an adjusted p value below 0.01. The adjusted p value is obtained from a t-test. Raincloud plots illustrate the boxplots, violin plots, and jitter plot of the log10 transformed delay values for each category.

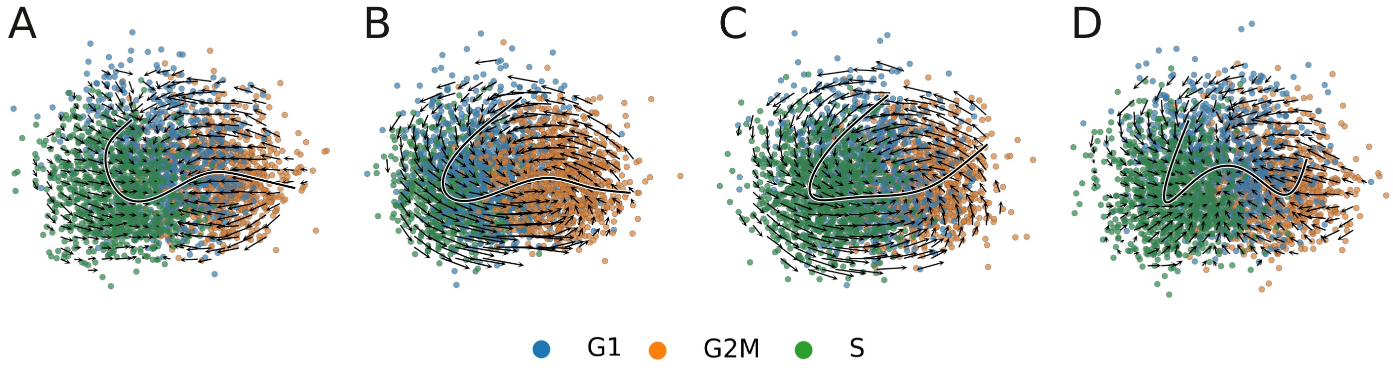

Figure S9
The velocity fields for all four technical replicates of the 293T cell line, the field represents the different phases of the cell cycle and the expected mRNA direction based on gene expression. Blue indicates G1 phase cells, green is for S phase, and orange is for G2/M. The principal curve shows the overall directionality of the data points. Each point in the velocity field represents an individual cell, the colors are illustrate the cell cycle phase of that cell.

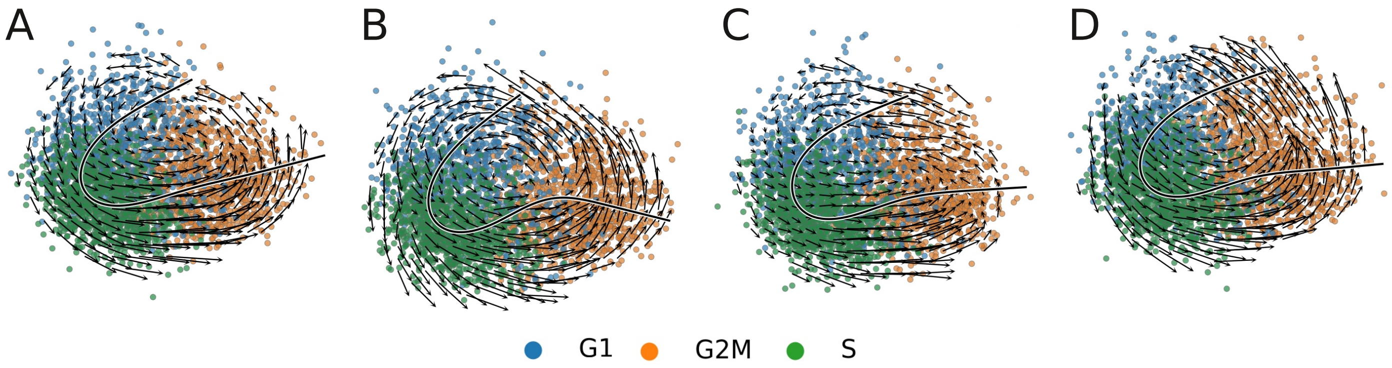

Figure S10
The velocity fields for all four technical replicates of the jurkat cell line, the field represents the different phases of the cell cycle and the expected mRNA direction based on gene expression. Blue indicates G1 phase cells, green is for S phase, and orange is for G2/M. The principal curve shows the overall directionality of the data points. Each point in the velocity field represents an individual cell, the colors are illustrate the cell cycle phase of that cell.

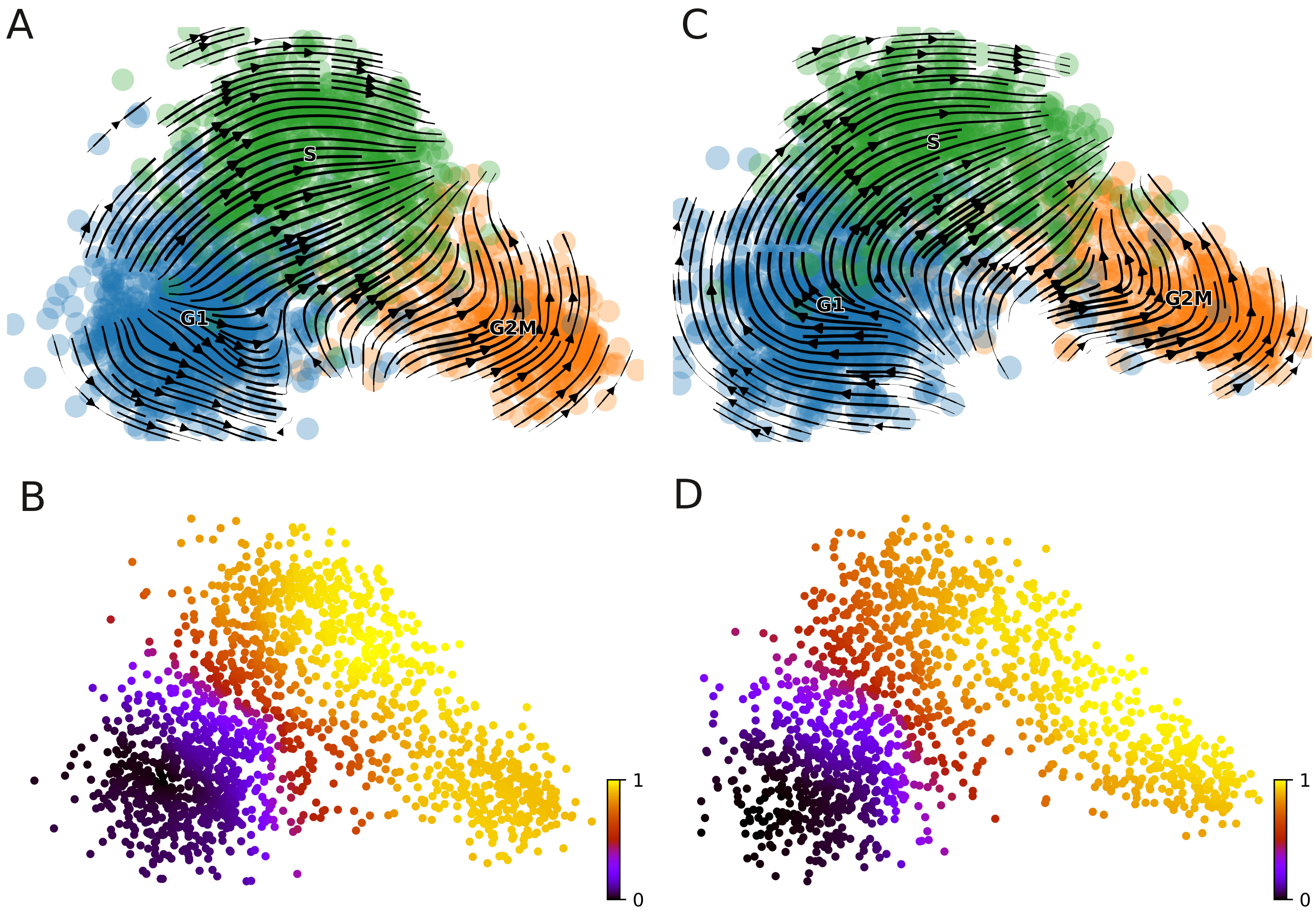

Figure S11
Results for the scVelo analysis on both HaCaT replicates, with the velocy field on the top row and the latent time analysis on the bottom. The first column shows replicate A (figures A and B) while the second (figures C and D) shows replicate B.

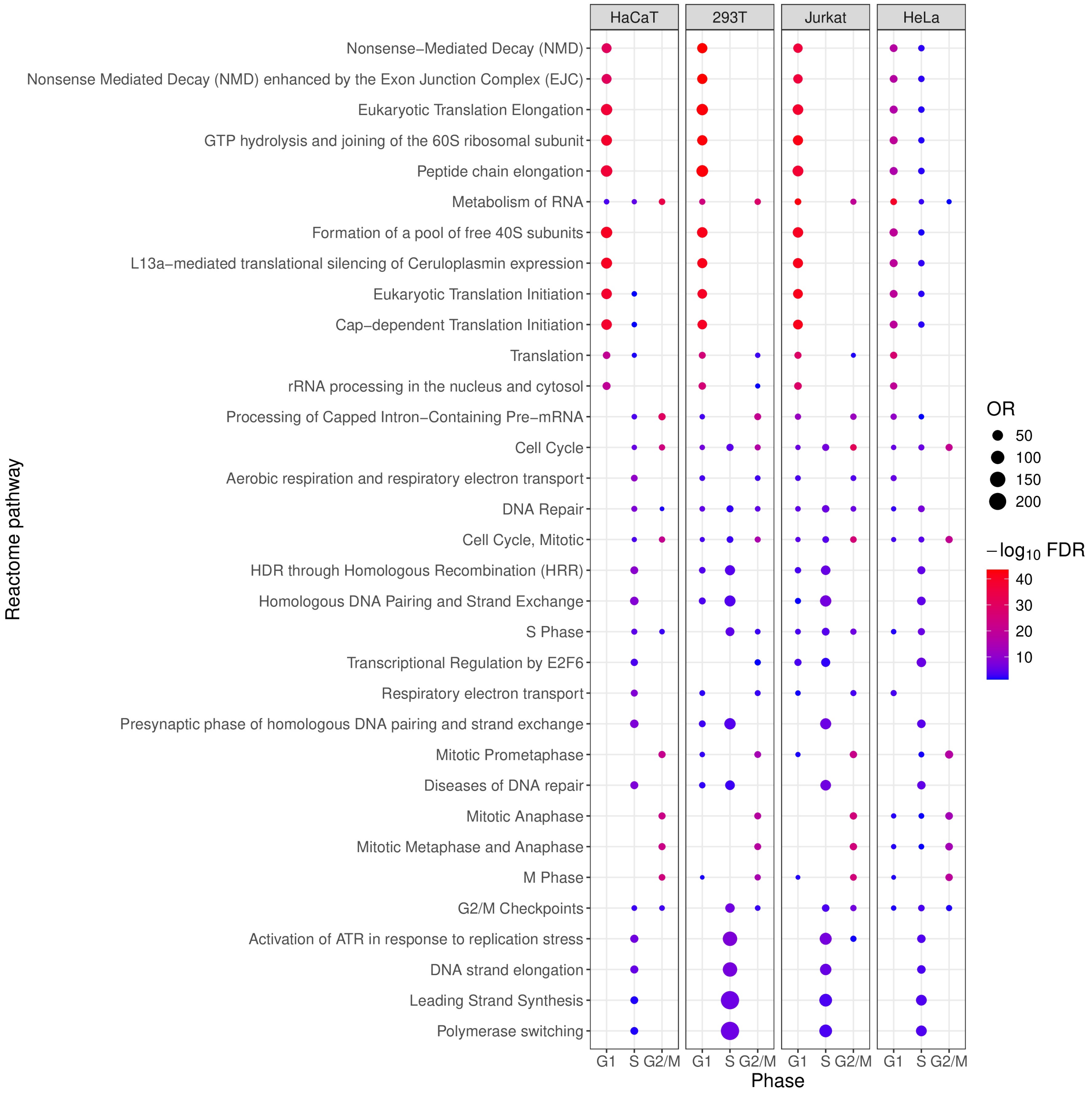

Figure S12

A dotplot showing REACTOME terms found for all four cell lines (HaCaT, 293T, Jurkat, HeLa), with a maximum of 5 terms per category on the x axis, top terms are selected based on false discovery rate (FDR) and the odds ratio (OR). The x axis shows the cell cycle phases. Term names are shown on the y axis. The size of each dot is proportional to the OR associated term on the y axis. The color scale is created using a -log10 transformation of the FDR which was calculated using the Benjamini-Hochberg approach.

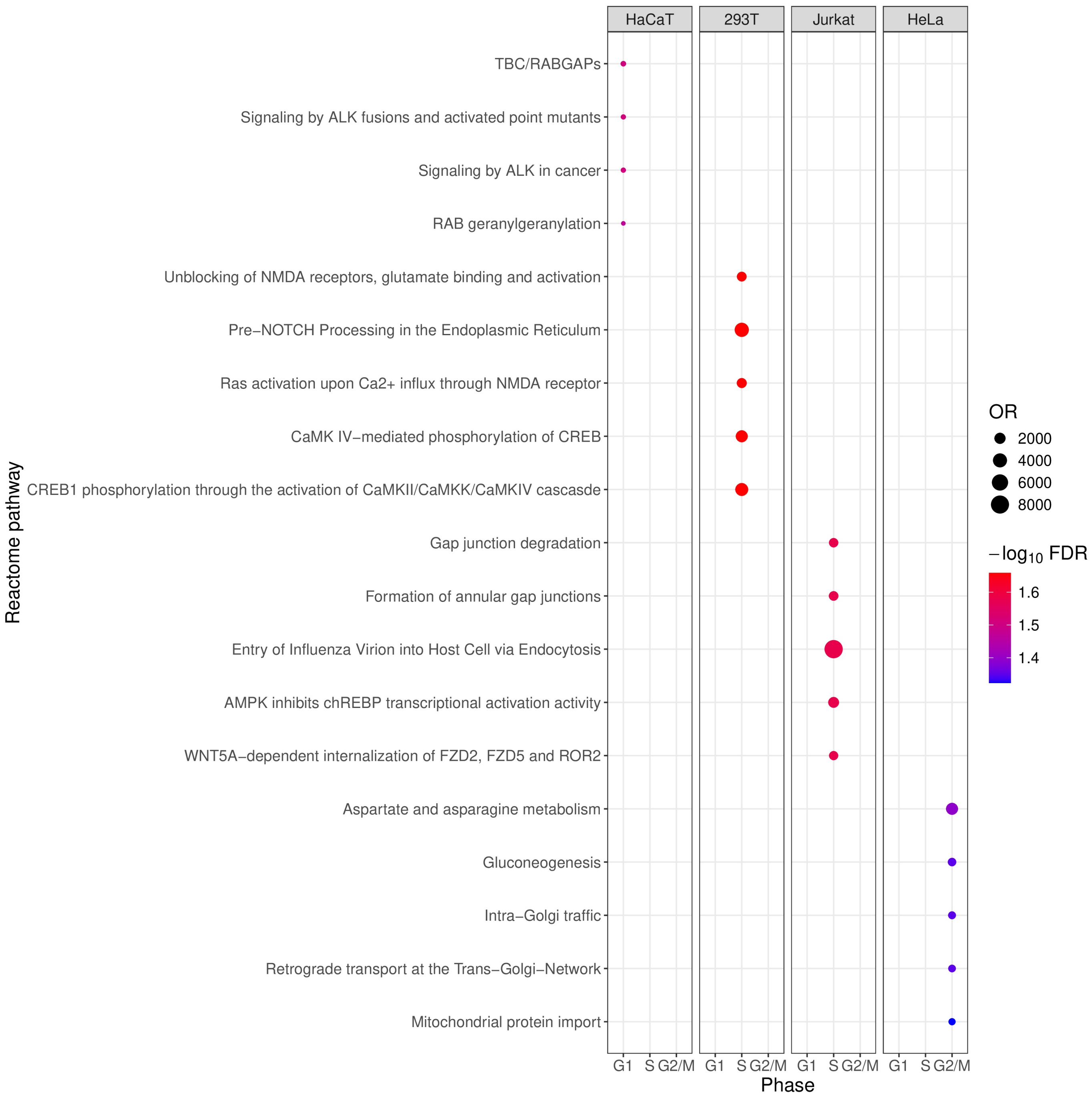

Figure S13

A dotplot showing REACTOME terms found for the HaCaT, 293T, Jurkat, and HeLa cell line using genes which were not considered to be significant in the respective cell line. A maximum of 10 terms are shown per cell cycle phase, top terms are selected using the faslse discovery rate (FDR) and the odds ratio (OR). The x axis shows the cell cycle phases. Term names are shown on the y axis. The size of each dot is proportional to the OR of the associated term on the y axis. The color scale is created using a -log10 transformation of the FDR which was calculated using the Benjamini-Hochberg approach.

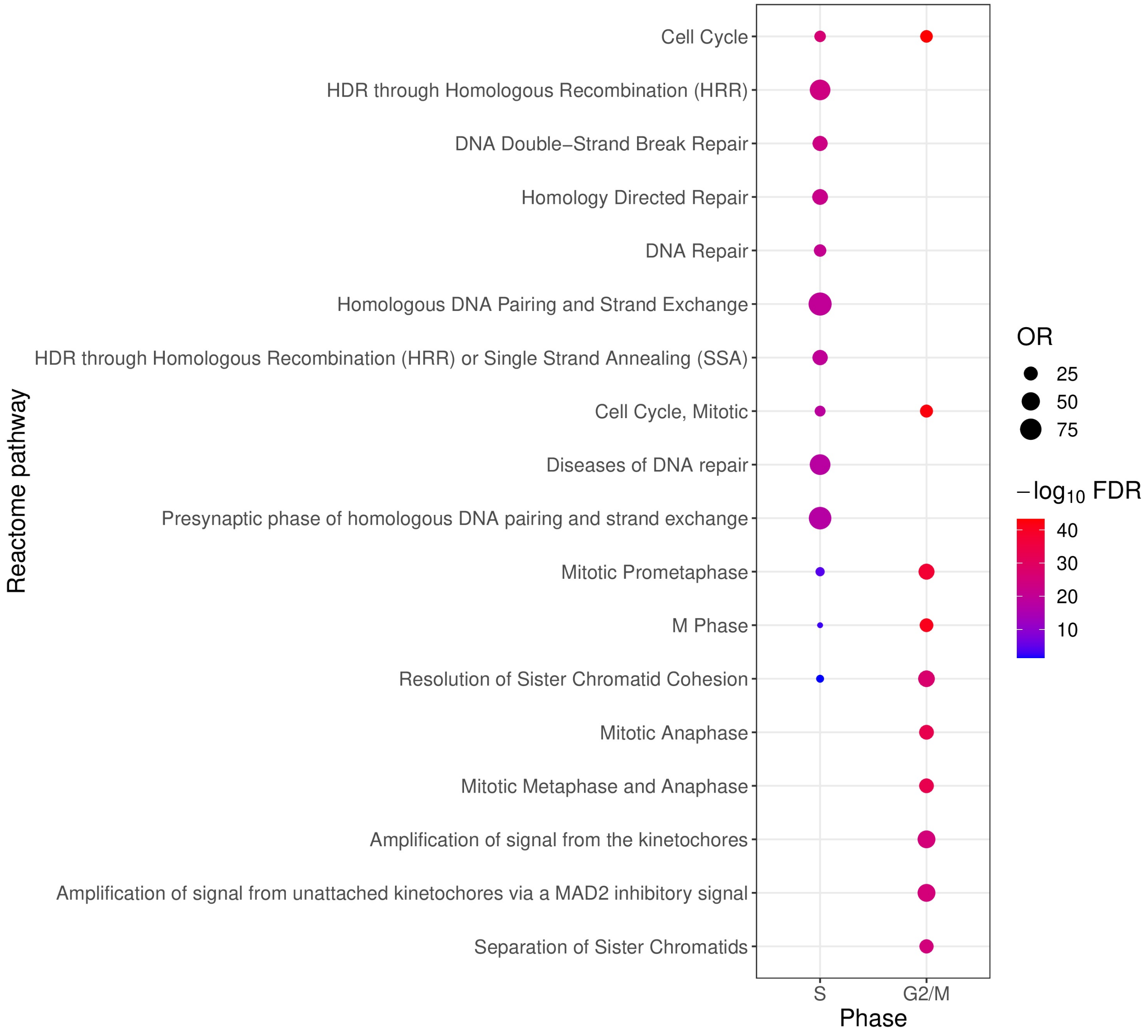

Figure S14

A dotplot showing REACTOME terms found for the HaCaT cell line following a cell cycle variance filter, and a filter based on negative delay, with a maximum of 10 terms per category. Top terms are selected based on false discovery rate (FDR) and the odds ratio (OR). The x axis shows the cell cycle phases. Term names are shown on the y axis. The size of each dot is proportional to the OR of the associated term on the y axis. The color scale is created using a -log10 transformation of the FDR which was calculated using the Benjamini-Hochberg approach.

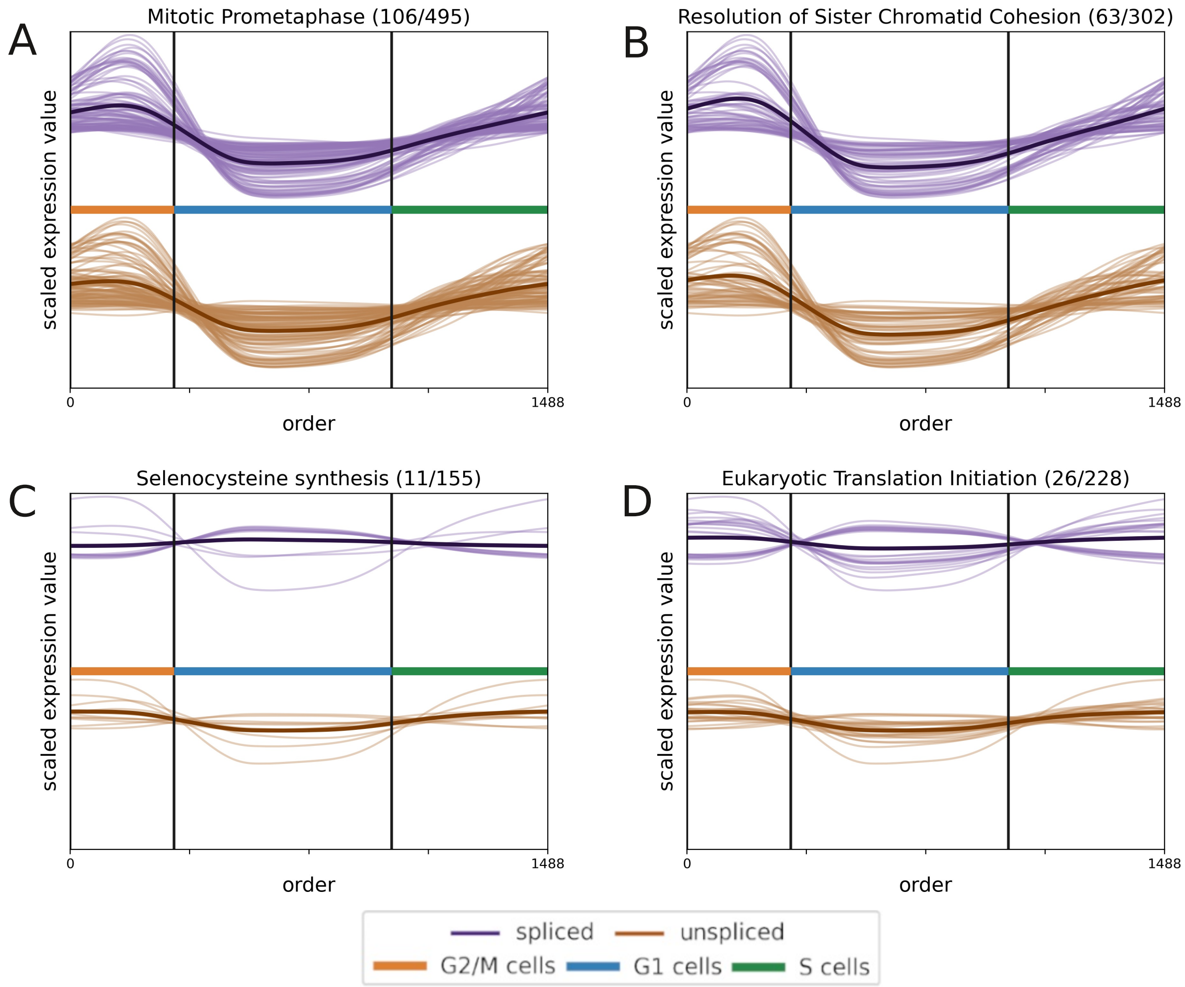

Figure S15
Line plots for the HaCaT cell line showing the trajectories of the spliced and unspliced expression values for genes of specific REACTOME pathways. Genes with an adjusted p value below 0.01 and a log10 cell cycle variance greater than the mean of the median varibility between each replicate were plotted. Spliced values are in purple and unspliced values are in orange. The mean trajectory is shown using a darker, thicker line in the plots. The values of the genes have been scaled. Cell cycle phases are indicated by the coloured bar in the middle of each graph, where orange represents G2/M, blue is G1, and green is S. The number of genes plotted along with the total number of genes in the REACTOME pathway are shown in the plot titles.

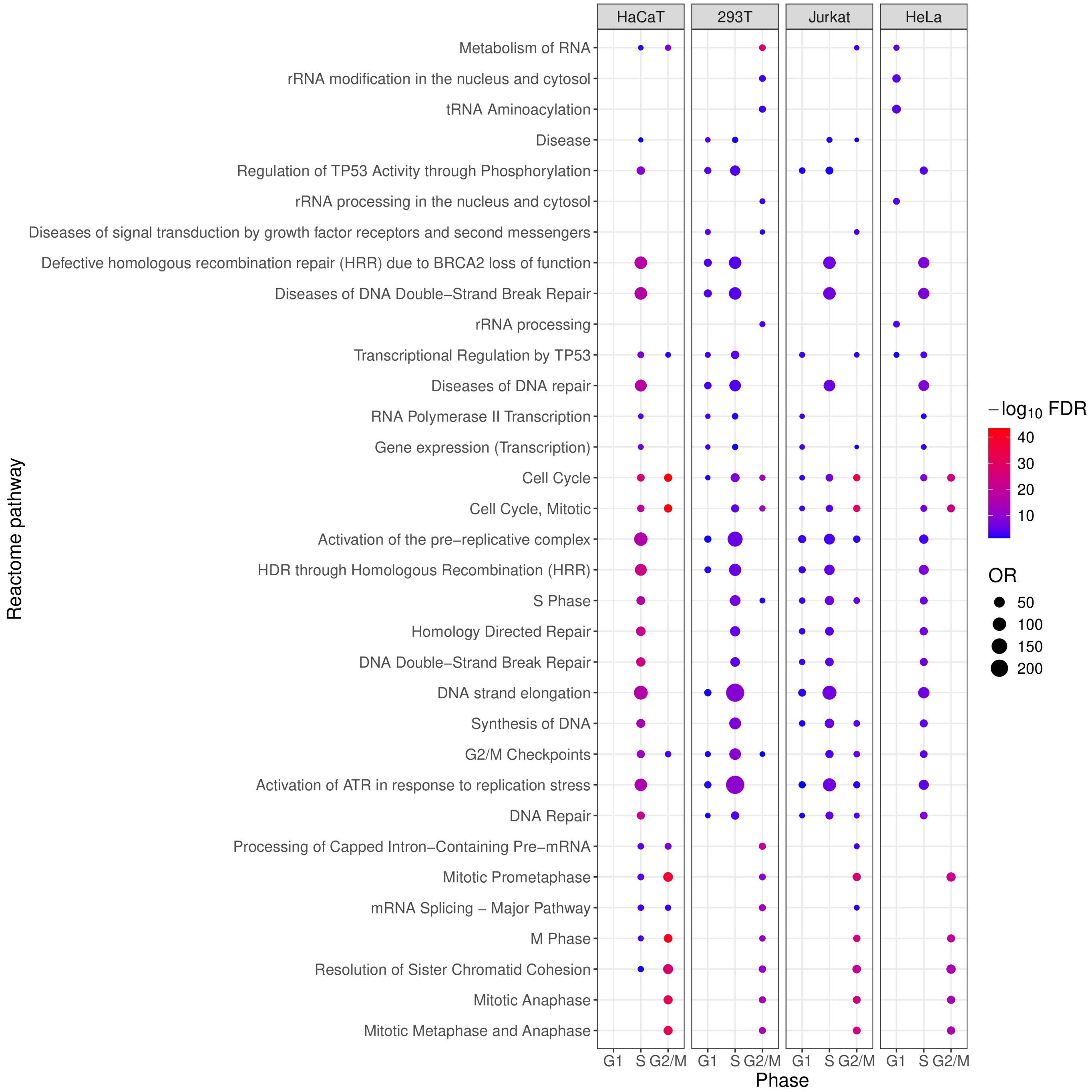

Figure S16

A dotplot showing REACTOME terms found for all four cell lines (HaCaT, 293T, Jurkat, HeLa) following a cell cycle variance filter, and a negative delay filter, with a maximum of 5 terms per category on the x axis, top terms are selected based on false discovery rate (FDR) and the odds ratio (OR). The x axis shows the cell cycle phases. Term names are shown on the y axis. The size of each dot is proportional to the OR of the associated term on the y axis. The color scale is created using a -log10 transformation of the FDR which was calculated using the Benjamini-Hochberg
