# Supplementary material for: Using pseudotime derivative on single-cell RNA sequencing data to identify genes undergoing cell cycle regulation
Source: Bioinform Adv. 2025 May 29;5(1):vbaf123. doi: 10.1093/bioadv/vbaf123 (PMC12255884; doi:10.1093/bioadv/vbaf123)
Supplement: vbaf123_Supplementary_Data [file vbaf123_supplementary_data.zip › vbaf123_AuthorCorr_CmtAttachmentsFolder_supplementary_file_1 (1) (2).pdf]

Using pseudotime derivative on single cell RNA sequencing data to identify genes undergoing cell cycle regulation - Materials and Methods

Yohan Lefol,[1,2,3,4,*] Geir Amund Svan Hasle,[2,5] Siv Anita Hegre,[2] Helle Samdal[2] and Pål Sætrom[2,5,6,7]

[1]Génie physiologique, biotechnologique et informatique, Université de Poitiers, 8600, France, [2]Department of Clinical and Molecular Medicine, NTNU — Norwegian University of Science and Technology, NO-7491, Norway, [3]Department of Microbiology, Institute of Clinical Medicine, University of Oslo, NO-0373, Norway, [4]CRESCO, Centre for Embryology and Healthy Development, University of Oslo, NO-0373, Norway, [5]St. Olavs hospital HF, Sentral Stab, NO-7006, Norway, [6]Department of Computer Science, NTNU — Norwegian University of Science and Technology, NO-7491, Norway and [7]K.G. Jebsen Center for Genetic Epidemiology, NTNU — Norwegian University of Science and Technology, NO-7491, Norway

*Corresponding author. yohanl@medisin.uio.no

Materials and Methods

Detailed below is the methodology for the creation of the two HaCaT technical replicates, their processing (from library preparation, sequencing, to demultiplexing and annotation). The specific quality control parameters are detailed followed by the protocol to the establishment of the cell cycle pseudotime order. Based on this order, we detail the means which were used to reassign cells to cell cycle phases as well as assigning them an order from a starting point within the cell cycle pseudotime. We then demonstrate the methods with which we merge technical replicates, with the product of the merger, we detail the means with which RNA velocity was calculated for individual genes. Finally, the various statistics involved are demonstrated, beginning with the t-test to calculate gene significance, the calculation of 'gene delay', the log10 variance filtering, gaussian mixuture modelling and delay thresholding as well as the differential gene expression analysis performed on the TCGA database along with the various statistical correlation tests that were performed with, and without the TCGA data.

All python packages used were downloaded using miniconda 23.1.0, the specific version of the main packages used are mentioned in the text below, all other packages used as well as their version are detailed on the github for this tool `https://github.com/Ylefol/CC_vel`.

Cell cultures

Both HaCaT and MEF cell lines were obtained from the American Type Culture Collection (ATCC) and cultivated in a humidified incubator at 37°C in 5% CO_2 . HaCaT is a human keratinocyte cell line and was cultured in Dulbecco's modified Eagle's medium (DMEM) (Sigma-Aldrich, D6419) supplemented with 10% fetal bovine serum (FBS) (Sigma-Aldrich, F7524), 0.1 mg/ml gentamicin (Gibco, 15710049), 2 mM L-Glutamine (Sigma-Aldrich, G7513) and 1.25 µg/ml fungizone (Sigma-Aldrich, A2942). MEF is a mouse embryonic cell line and was cultured in DMEM supplemented with 10% FBS, 1 % Penicillin-Streptomycin (Sigma-Aldrich, P0781), 2 mM L-Glutamine and 1 % MEM non-essential amino acid solution (Sigma-Aldrich, M7145). We harvested the cells at about 70 % confluency and counted them using the Moxi Z cell counter (ORFLO Technologies). We made a suspension of 900 000 HaCaT cells and 100 000 MEF cells and centrifuged at 150 relative centrifugal force (rcf) for 5 minutes at room temperature (RT). After washing the cells by carefully resuspending them in 1 ml phosphate buffered saline (PBS) (Sigma-Aldrich, BR0014G) supplemented with 400 µg/ml Bovine Serum Albumin (BSA) (Sigma-Aldrich, A7030), we counted and centrifuged the cells at 150 rcf for 5 minutes at RT. Then PBS-BSA solution was added to achieve a cell concentration of about 1200 cells per µL, followed by gentle resuspension of the cells before they were transferred through a 40 µM cell strainer. Cell suspension was kept on ice until proceeding with the 10x Genomics® Single Cell Protocol.

Library preparation and sequencing

Cells were processed using the 10x Genomics Chromium Controller and the Chromium Single Cell 3' Gene Expression Library and Gel Bead Kit 2 following the standard manufacturer's protocols. Between 6000 and 8000 live cells were loaded onto the Chromium controller in an effort to recover between 3000 and 4000 cells for library preparation and sequencing. Gel beads were prepared according to standard manufacturer's protocols. Oil partitions of single-cell oligo coated gel beads (GEMs) were captured and reverse transcription was performed, resulting in cDNA tagged with a cell barcode and unique molecular identifier. GEMs were then broken and cDNA was amplified and quantified using an Agilent Bioanalyzer High Sensitivity chop (Agilent Technologies). To prepare the final libraries, amplified cDNA was enzymatically fragmented, end-repaired, and polyA tagged. Fragments were then size selected using SPRIselect magnetic beads (Omega Bio-tek). Illumina sequencing adapters were then ligated to the size-selected fragments and cleaned using SPRIselect magnetic beads. Lastly, sample indices were selected and amplified, followed by a double sided size selection using SPRIselect magnetic beads. Final library quality was assessed using an Agilent Bioanalyzer High Sensitivity chip. Samples were then sequenced on the Illumina NextSeq 500 with 26 cycles for Read 1, 8 cycles index read and 134 cycles for Read 2.

Public datasets

The 293T and Jurkat data were produced by Zheng et al and stored on short read archive, accession number SRP073767 (Zheng et al., 2017). For this manuscript, the data was downloaded from the 10x website: `https://support.10xgenomics.com/single-cell-gene-expression/datasets/1.1.0/293t` and `https://support.10xgenomics.com/single-cell-gene-expression/datasets/1.1.0/jurkat`.

Demultiplexing

Demultiplexing was done using CellRanger 3.0.0 with the mkfastq workflow. The mkfastq workflow reads the Illumina sequencer output files and generates fastq files for each of the samples. The fastq files were organized as two files, one for read one (R1) and another for read two (R2). The R1 files contain the barcode reads and the unique molecular identifier reads, while R2 consists of the corresponding cDNA reads. Two technical replicated of the HaCaT-MEF culture are sequenced, which creates two sets of R1 and R2, named HaCaT_MEF_A and HaCaT_MEF_B accordingly. For the 293T and Jurkat dataset the fastq files had to be reformatted to utilize CellRanger as these two cell lines were processed with 10x version 1 chemistry, which is not directly compatible with current versions of CellRanger.

Demultiplexing is performed with the cellranger count workflow using a reference index built with the recipe from the 10X ressource page (`https://support.10xgenomics.com/single-cell-gene-expression/software/release-notes/build#hg19mm103.0.0`), with the expection that release 94 of GRCh38 will be used instead of release 93. The count matrices are converted to a .h5ad AnnData object and loaded into SCANPY. Cell barcodes with 0 reads are removed as well as all genes that have 0 reads. All genes are either mapped to humans or to mice and labelled accordingly using the prefixes of GRCh38_ and GRCm38_. Cells with more than 15% murine genes are considered to be a murine cell, cells below the threshold are considered to be human. Mouse and human barcodes are stored in text files for later use and demultyplexed data is run through the Velocyto command line tool (CLI).

Annotation of spliced and unspliced reads

The velocyto.py CLI was used for read annotation. A demultiplexed cell-specific BAM file similar to a Cellranger output is fed into velocyto.py along with the GRCm38 and GRCh38 gene annotation files for the murine and human cells respectively. The gene annotation files are used to count molecules in each cell and sort them as 'spliced', 'unspliced', and 'ambiguous'. All molecules must be uniquely mapped to be accepted. Cases where the transcript can be associated with two or more genes are rejected.

The Velocyto CLI was run using the snakemake rules (Köster and Rahmann, 2012) outlined on the genome core facility's github (gcfntnu) `https://github.com/gcfntnu/single-cell/blob/master/rules/quant/velocyto.rules`.

Dataset Quality control

Both technical replicates undergo a series of quality controls in which unnecessary cells are removed along with genes and cells with abnormal reads. We first remove murine cells from our dataset using specific cell barcodes associated to the murine cells. Afterwards we remove cells which contain less than 500 unique mRNA reads as well as cells containing less than 4000 overall mRNA reads. Genes which are expressed in less than 5 cells are also removed. We then check the percentage of mitochondrial reads per cell to figure out which percentage threshold should be utilized, following the model detailed by SCANPY, version 1.5.1, which is strongly inspired by Seurat (Satija et al., 2015). We used PyRanges, version 0.0.79 to identify all mitochondrial genes from a GTF file, the list of mitochondrial genes served as the base to the calculation of the percentage of mitochondrial reads (Stovner and Sætrom, 2019). Following the finding of the threshold (0%), we filter out any cells with a mitochondrial percentage above 0%. We then filter out cells with too high or low, unspliced reads in contrast to spliced reads, for this filter a maximum percentage threshold of 25% was used, where if 25% or more of the reads in a cell were unspliced, the cell is removed from the dataset. The lower filter was 10%. The last step of the quality control is to remove any genes that have no unspliced reads associated to them. Thresholds for the 293t and Jurkat cell line were 10%, 6% for the maximum and minimum unspliced thresholds respectively, and a 0% mitochondrial threshold.

Establishing a pseudotime order

Briefly, pseudotime order was determined by running a principal component analysis (PCA) on a subset of the count matrix containing 172 genes with known cell cycle-dependent expression (Whitfield et al., 2002; Tirosh et al., 2016) and using the cells' phase angle in the plane defined by principal components (PCs) 1 and 2 as the order. Specifically, following quality control, effects of varying sequencing depth per cell were removed by running the ScanPy regress_out function on the count matrix with the number of reads per cell before normalization (n_counts) as the variable to regress on. The resulting residual matrix was filtered by removing all genes except any of the 172 known cell cycle genes and the filtered residual matrix was used to run a PCA. Each cell was then assigned a phase angle by calculating the arctangent of the cell's PC1 and PC2 and adding 2π to the result for cells with negative arctangent values.

Assigning cell cycle phases

Briefly, each cell was assigned to one of the cell cycle phases G1, S, and G2/M by using the phase angle distributions of cells predicted to be in S or G2/M to identify the most likely phase angles separating the G1, S, and G2/M phases in the pseudotime order. Specifically, phase scores were first assigned to each cell by comparing the average expression of three sets of G1, S, and G2/M genes with an average reference expression (Wolf et al., 2018). Second, we selected the top 10% scoring cells for each phase as the phase's representative cells and removed from these the 10% cells closest to the origin in the PC1/PC2 plane of the pseudotime order PCA. Third, for the set of cells in each of the S and G2/M phases, we computed the set's mean phase angle and standard deviation and used these to construct a normal distribution-based phase angle probability density function for the S and G2/M phases (Pena-Diaz et al., 2013). Means and standard deviations were computed both for the initial angles (i.e. μ, σ) and for angles rotated by π (i.e. μ^r, σ^r) to get accurate parameter estimates for phases with cells clustered around phase angle $0/2\pi$. Specifically, if $\sigma^r < \sigma$ we used $\mu^r - \pi$ ($\mu^r + \pi$ if $\mu^r < \pi$) and σ^r as the phase's mean and standard deviation. Fourth, as cells assigned to G1 tended to span the entirety of the PCA plot, we used a kernel density estimate of the G1 phase angles as the G1 phase probability

density function. We identify the largest phase angle region where the G1 distribution is higher than the S and G2/M distribtions as the G1 region and use the mean phase angle of G1 cells within that region as the mean G1 angle. Finally, the start and end angles of the G1 region define the G1 - S and G2/M - G1 boundries, whereas the angle where the S and G2/M phase probability density functions are equal define the S - G2/M boundry.

Assigning pseudotime zero

We used the phase angle for the S - G2/M boundry to define pseudotime zero. In contrast to the G1 cells, S and G2/M cells tended to cluster, resulting in the boundry between S and G2/M being more well defined than the other two boundries. One possible explanation is that for both the pseudotime order PCA and the phase predictions, the gene sets used are dominated by genes expressed in S or G2/M.

Starting at pseudotime zero, cells were then assigned pseudotimes by order of increasing or decreasing phase angles depending on whether the order of the mean phase angles were G2/M-G1-S or G2/M-S-G1, respectively. PCA can produce both orderings, but for the G2/M-S-G1 ordering, the order of the phase angles must be reversed for the resulting pseudotime to be consistent with the cell cycle.

Assigning cell cycle phases with DeepCycle

We used the standard DeepCycle pipeline (Riba et al., 2022) to process the HaCaT replicates. The miniconda environment was build according to their documentation found on their github (`https://github.com/andreariba/DeepCycle/`). We add scVelo (version 0.2.5) (Bergen et al., 2020) to the DeepCycle miniconda environment in order to process our loom files with the pp.moments function as required by the DeepCycle documentation. We the utilize the 'DeepCycle.py' script with hotelling turned off followed by the 'estimate_cell_cycle_transitions.py'. The results are saved in an h5ad file compatible with other elements of this method, such as calculating gene specific velocity.

Characterizing G1 infiltrators

The identifier for G1 cells found within the boundaries of the S and G2M phases were identified and saved as 'G1_in_S' and 'G1_in_G2M' respectively. We then utilized scanpy's 'rank_genes_groups' to compare these groups with the G1 cells within the G1 boundaries. Significant genes were marked as having a pvalue below 0.01 and an absolute fold change greater than 1. We then took the upregulated genes (positive fold change) and submitted them to an over representation analysis via Gprofiler (Raudvere et al., 2019) of which the top 20 REACTOME results were saved.

Gene specific velocity: filtering and normalization

Velocyto (v.017.17) was modified to read loom files which, following an update from the loompy package, no longer followed the expected naming convention. We perform a gene filter requiring that each gene must have a minimum of 30 reads for both unspliced and spliced and must be expressed by at least 10 cells. We then proceed to downsample the cell amount in order to balance the amount of cells associated to each cell cycle phase, the cells filtered out are selected randomly. This is followed by a size normalization on both the spliced and unspliced layers.

From here the standard Velocyto workflow is followed to generate the velocity field, this implies performing a k-nn smoothing of the data matrix using a k of 550 followed by normalizing the median (normalizing cell sizes to the median size based on both spliced and unspliced values). Following this we fit the gamma distribution used to both the spliced and unspliced data. The data is normalized once again using the 'inputted' parameter. The unspliced data for the gamma fit prediction is generated by multiplying the value of the gamma fit with the value of the 'Sx' at each time point. Sx is defined by the Knn smoothed expression data.

Velocity fields

We then calculate the cell velocities and the shift, which corresponds to the change in gene expression for every cell. Using the shift, we extrapolate the gene expression profile for each cell at a specified time step using increments of 1. The estimation of transition probability is done for every cell for it's embedding neighbour using correlation. For plotting purposes, we use transition probability to project the velocity's direction onto the embedding, the grid arrows are then calculated using a point on a regular grid along with a gaussian kernel. These steps are finalized by plotting the velocity field (velocity arrows on top of the PCA plot). The velocity field is enhanced by adding a principal curve via the 'princurve' R package (Hastie and Stuetzle, 1989), this provides a non-linear summary of the data. The principal curve is fitted using the principal components used in the velocity field, in our case this represents PCs 1,2, and 3.

Gene specific velocity: Computing velocities

By utilizing the angle boundaries previously calculated, we determine the exact cells that represent the beginning of each phase, this is then used in plots to represent the different phases of the cell cycle via horizontal colored bars.

To determine the specific velocity of each gene we calculate the smoothed means for both the unspliced and spliced values using a bin size of 100 data points by using NumPy's mean function with intervals of 100. Resulting datapoints preserve their point in time and are then elongated to fit the size of the dataset. We then further smooth the data with a moving average. Let X be the data points, M be the window size, n be the size of the sample, and $\overline{a}_{SM}$ be the calculated average. Using equation 1 we calculate the average for each datapoint using a window size equal to 20% of the amount of cells, and moving the window forward by one point at a time, as the window is moving forward, the full summation is not required after every movement, we thus calculate the first value with equation 2, and move along the dataset by increments of 1.

$$\overline{a}_{SM} = \overline{a}_{SM\_prev} + \frac{1}{n}(x_M - x_{M-n}) \tag{1}$$

$$\overline{a}_{SM\_prev} = \frac{x_n + x_{n-1} + \ldots + x_{M-(n-1)}}{M} \tag{2}$$

We then calculate the derivatives of the resulting values using Numpy's diff function, which implies the calculation of the n-th discrete difference along the axis. Following equation 3 where y represents our smoothed expression values and x represents our pseudotime (i.e. the cells in the dataset from 1 to n), we obtain the slope for the velocity.

$$m = \frac{dy}{dx} \tag{3}$$

Gene specific velocity: Computing confidence intervals and replicate merger

All steps previously detailed in this section are performed 5 times per replicate, we then merge all iterations/replicates, in the case of the HaCaT cell line, two replicates, with 5 iterations each. Replicates are merged by first identifying the smallest replicate in regards to the number of cells. All replicates are balanced in accordance to the size of the smallest replicate, the appropriate number of cells are removed from larger replicates, removed cells are evenly spaced out throughout the replicate. In addition, only genes which are found in all submitted replicates are preserved. This results in replicates which are of the same size (number of cells) and genes. We then calculate the mean expression and velocity of each gene along with the 99% confidence interval.

Gene specific velocity: Simplifying gene velocity

Based on the velocities and the confidence intervals we reduce a genes velocity to +1,-1, or 0. A value of +1 is given when both the velocity and the lower confidence interval show positive values, a value of -1 is the opposite. A value of zero is given when the velocity and the confidence intervals do not agree on the direction of the velocity (one shows a positive value while the other a negative). Put together, this format provides a simplified view of when velocity is positive, negative or uncertain.

Calculating significance of genes

Genes are considered for ranking only if the confidence intervals confirm both a positive and negative velocity for both the unspliced and spliced curves. If this condition is met, a score is given to the four categories of the gene (positive and negative velocity for spliced and unspliced). The score is calculated from the product of the merger (see Gene specific velocity: Computing confidence intervals and replicate merger). During the merging of the replicates we obtain the mean velocities of genes as well as the standard deviations for each gene at each time point/cell in the pseudotime. We then identify the cell which fits the category being tested, for example, if we seek to score the positive spliced velocity of geneX we will look for the cell whose spliced velocity is highest in for geneX, we refer to this cell as 'target_peak' as it represents the highest point (peak) of spliced velocity. We then calculate a z-score for geneX by dividing the value of the target_peak with the standard error of the mean, see equation 4. The score therefore serves as a ranking of genes in addition to be used for a t-test of significance (see below).

$$score = \frac{target\_peak}{(\frac{\sigma}{\sqrt{number\_of\_iterations}})} \quad (4)$$

Once a score has been calculated for every gene, we submit these scores to a student's t-test with R's pt function, where lower.tail=FALSE. The hypothesis of this test is whether or not the mean of the scores are different from 0. The pvalue is then calculated using a two-tailed hypothesis approach. The adjusted p-value is calculated using the p_adjust function in R with FDR (False Discovery Rate) as the method used.

Alternative method: Extreme value testing

As we are testing the peaks (or valleys) of the genes' velocity, a more stringent approach to calculating gene significance is to use generalized extreme value distribution. Specifically, for a given gene, we could rest whether the observed maximum (or minimum) velocity is more extreme than those observed in random data. To implement this idea as a statistical test, we for each gene, generated ten random velocity peaks, fitted a generalized extreme value (GEV) distribution to the random velocity peaks from all genes, and used the cumulative distribution function for the fitted GEV to compute the p-value for each gene. That is, given the GEV cummulative distribution F_X and the gene with peak value x, the p-value for the gene's was $1 - F_X(x) = 1 - P(X \leq x)$. Note that to preserve the correlation structure of the single cell data we generated the random peaks by randomly rotating the velocity profiles from the replicate experiments from each gene and then computed the maximum peak in the combined rotated data.

Calculating gene delay

Using the +1,-1,0 velocity format, we sort genes by delays into four categories: $0 \rightarrow 1$, $1 \rightarrow 0$, $0 \rightarrow -1$, and $-1 \rightarrow 0$. With the first two categories representing positive velocities, hence active transcription, and the last two categories represent the opposite. We first find a single point in the data where the symbols match (both the velocity and confidence interval show either positive or negative values) and 'roll' the data to set the zero point as the found location, this ensures that all differences are located within the window and will not overlap with the zero point. The purpose of delays is to measure the time (along the pseudotime axis) it takes for unspliced and spliced values to match. Primarily, the intent is to observe the time between a 'prediction' event (a change in velocity of unspliced mRNA) to be matched by the velocity of spliced mRNA. Therefore, delays are calculated using the delta of the pseudotime ordering of a change in spliced and unspliced. This results in a positive delay for 'prediction' events, and a negative delay for instances where a changed in spliced mRNA precedes unspliced mRNA. Delays are measured for each of the four categories, therefore a single gene will have four delays associated to it, one per category.

Calculating the negative gene delay threshold

Using the delay values from the decrease to 0 delay category, representing a genes transition from peak velocity to 0 velocity/peak expression, we used sklearn's (Pedregosa et al., 2011) gaussian mixture modelling to separate a trimodal distribution into three distinct distributions (setting the 'n_components' and 'covariance_type' parameters to 3 and 'tied' respectively). Additionally, we set the seed (random_state) to '123' to ensure reproducibility. Three distinct curves are obtained, with one having a mean of, or close to 0. This curve is then utilized with scipy's ppf function to identify the 95th percentile on the lower tail, resulting in the negative delay threshold.

Calculating cell cycle variance

Cell cycle variance was obtained by log10 transforming gene expression data for genes contained within selected REACTOME pathways. For each pathway, the variance throughout the pathway is calculated using the .var function within the pandas library. We then calculate the median of each pathway, and the mean of that median is used as the cell cycle variance threshold.

TCGA differential gene expression analysis

R (version 4.2.2) was used in conjunction with TCGAbiolinks (Colaprico et al., 2016) (version 2.25.3) and limma (Ritchie et al., 2015) (version 3.54.1) to produce a list of significant upregulated genes for all cancers contained within the TCGA database. TCGA data was downloaded for all cancers (BRCA, OV, LUAD, UCEC, GBM, HNSC, KIRC, LGG, LUSC, THCA, PRAD, SKCM, COAD, STAD, BLCA, LIHC, CESC, KIRP, SARC, ESCA, PAAD, READ, PCPG, TGCT, LAML, THYM, ACC, MESO, UVM, KICH, UCS, CHOL, and DLBCL). This was done using TCGAbiolinks by querying for a vector containing the cancer acronyms while setting the data.category to 'Gene expression' and data.type to 'Gene expression quantification'. The platform was declared as 'Illumina HiSeq' and the file.type to 'normalized_results', legacy was set to TRUE. We then used the TCGAanalyze_Filtering function with the 'quantile' method and a qnt.cut of 0.25. Tumor (TP) and non-tumor (NT) were identified. We then used the voom function (Law et al., 2014) within limma followed by a simple design matrix of '∼0 + tumorType' (comparing non-tumor vs tumor). The standard limma protocol was followed, therefore we fit the data using lmFit, followed by constrast.fit to create the necessary contrasts, and finally the eBayes function to calculate the statistics. Significant genes were extracted, with significant genes being considered as having an adjusted pvalue below 0.05 and an absolute log fold change greater than 1. Upregulated genes were isolated by extracting all significant genes with a log fold change greater than +1.

Statistical comparisons

Correlation between gene delay categories representing active transcription and no transcription were performed using spearman's correlation. Both the active transcription and the no transcription categories are split into two delay categories: 'increase to 1' and 'decrease to 0' for active transcription and 'decrease to -1' and 'increase to 0' for no transcription. These sub categories were compared using the spearmanr function from the scipy package (Virtanen et al., 2020).

Gene overlap was compared using the chi-square approach. In a first case, the overlap of genes was performed between cell lines using the chi2_contingency function from the scipy package. Overlap was performed between cel lines again, this time splitting genes into subgroups of the cell cycle phase in which they are categorized based on either the location in which they obtain peak velocity, peak expression, or where velocity begins to indicate active transcription. The odds ratio was also calculated in context of the odds of a overlap of significant genes.

References

V. Bergen, M. Lange, S. Peidli, F. A. Wolf, and F. J. Theis. Generalizing rna velocity to transient cell states through dynamical modeling. *Nature biotechnology*, 38(12): 1408–1414, 2020.

A. Colaprico, T. C. Silva, C. Olsen, L. Garofano, C. Cava, D. Garolini, T. S. Sabedot, T. M. Malta, S. M. Pagnotta, I. Castiglioni, et al. Tcgabiolinks: an r/bioconductor package for integrative analysis of tcga data. *Nucleic acids research*, 44(8):e71–e71, 2016.

T. Hastie and W. Stuetzle. Principal curves. *Journal of the American Statistical Association*, 84(406):502–516, 1989.

J. Köster and S. Rahmann. Snakemake—a scalable bioinformatics workflow engine. *Bioinformatics*, 28(19): 2520–2522, 2012.

C. W. Law, Y. Chen, W. Shi, and G. K. Smyth. voom: Precision weights unlock linear model analysis tools for rna-seq read counts. *Genome biology*, 15(2):1–17, 2014.

F. Pedregosa, G. Varoquaux, A. Gramfort, V. Michel, B. Thirion, O. Grisel, M. Blondel, P. Prettenhofer, R. Weiss, V. Dubourg, J. Vanderplas, A. Passos, D. Cournapeau, M. Brucher, M. Perrot, and E. Duchesnay. Scikit-learn: Machine learning in Python. *Journal of Machine Learning Research*, 12:2825–2830, 2011.

J. Pena-Diaz, S. A. Hegre, E. Anderssen, P. A. Aas, R. Mjelle, G. D. Gilfillan, R. Lyle, F. Drabløs, H. E. Krokan, and P. Sætrom. Transcription profiling during the cell cycle shows that a subset of polycomb-targeted genes is upregulated during dna replication. *Nucleic acids research*, 41(5): 2846–2856, 2013.

U. Raudvere, L. Kolberg, I. Kuzmin, T. Arak, P. Adler, H. Peterson, and J. Vilo. g: Profiler: a web server for functional enrichment analysis and conversions of gene lists (2019 update). *Nucleic acids research*, 47(W1):W191–W198, 2019.

A. Riba, A. Oravecz, M. Durik, S. Jiménez, V. Alunni, M. Cerciat, M. Jung, C. Keime, W. M. Keyes, and N. Molina. Cell cycle gene regulation dynamics revealed by rna velocity and deep-learning. *Nature communications*, 13(1):2865, 2022.

M. E. Ritchie, B. Phipson, D. Wu, Y. Hu, C. W. Law, W. Shi, and G. K. Smyth. limma powers differential expression analyses for rna-sequencing and microarray studies. *Nucleic acids research*, 43(7):e47–e47, 2015.

R. Satija, J. A. Farrell, D. Gennert, A. F. Schier, and A. Regev. Spatial reconstruction of single-cell gene expression data. *Nature biotechnology*, 33(5):495–502, 2015.

E. B. Stovner and P. Sætrom. PyRanges: efficient comparison of genomic intervals in Python. *Bioinformatics*, 36(3):918–919, 08 2019. ISSN 1367-4803. doi: 10.1093/bioinformatics/btz615. URL `https://doi.org/10.1093/bioinformatics/btz615`.

I. Tirosh, B. Izar, S. M. Prakadan, M. H. Wadsworth, D. Treacy, J. J. Trombetta, A. Rotem, C. Rodman, C. Lian, G. Murphy, et al. Dissecting the multicellular ecosystem of metastatic melanoma by single-cell rna-seq. *Science*, 352 (6282):189–196, 2016.

P. Virtanen, R. Gommers, T. E. Oliphant, M. Haberland, T. Reddy, D. Cournapeau, E. Burovski, P. Peterson, W. Weckesser, J. Bright, et al. Scipy 1.0: fundamental algorithms for scientific computing in python. *Nature methods*, 17(3):261–272, 2020.

M. L. Whitfield, G. Sherlock, A. J. Saldanha, J. I. Murray, C. A. Ball, K. E. Alexander, J. C. Matese, C. M. Perou, M. M. Hurt, P. O. Brown, et al. Identification of genes periodically expressed in the human cell cycle and their expression in tumors. *Molecular biology of the cell*, 13(6):1977–2000, 2002.

F. A. Wolf, P. Angerer, and F. J. Theis. Scanpy: large-scale single-cell gene expression data analysis. *Genome biology*, 19:1–5, 2018.

G. X. Zheng, J. M. Terry, P. Belgrader, P. Ryvkin, Z. W. Bent, R. Wilson, S. B. Ziraldo, T. D. Wheeler, G. P. McDermott, J. Zhu, et al. Massively parallel digital transcriptional profiling of single cells. *Nature communications*, 8(1):14049, 2017.
